# Pfs230 domain 12 is a potent malaria transmission–blocking vaccine candidate

**DOI:** 10.1126/sciadv.adw8216

**Published:** 2025-11-07

**Authors:** Maartje R. Inklaar, Roos M. de Jong, Dari F. Da, Lisanne L. Hubregtse, Maartje Meijer, Karina Teelen, Ezra T. Bekkering, Sanne Grievink, Marga van de Vegte-Bolmer, Geert-Jan van Gemert, Rianne Stoter, Hikaru Nagaoka, Takafumi Tsuboi, Eizo Takashima, Cornelia G. Spruijt, Michiel Vermeulen, Roch K. Dabire, Emmanuel Arinaitwe, Anna Cohuet, Teun Bousema, Matthijs M. Jore

**Affiliations:** ^1^ Department of Medical Microbiology, Radboud University Medical Center, Nijmegen, Netherlands.; ^2^ Institut de Recherche en Sciences de la Santé, Direction Régionale, Bobo Dioulasso, Burkina Faso.; ^3^ Division of Malaria Research, Proteo-Science Center, Ehime University, Matsuyama, Japan.; ^4^ Division of Cell-Free Sciences, Proteo-Science Center, Ehime University, Matsuyama, Japan.; ^5^ Department of Molecular Biology, Faculty of Science, Oncode Institute, Radboud University Nijmegen, Nijmegen, Netherlands.; ^6^ Infectious Diseases Research Collaboration, Kampala, Uganda.; ^7^ MIVEGEC, Montpellier University, IRD, CNRS, Montpellier, France.

## Abstract

Malaria transmission–blocking vaccines (TBV) target sexual stage parasites that are transmitted to mosquitoes and are critical for spread of the pathogen. The clinically most advanced TBV candidate contains part of the Pro-domain (Pro) and Domain 1 (D1) of *Plasmodium falciparum* surface protein Pfs230. Subunit vaccines that contain other domains of Pfs230 have so far failed to induce functional antibodies. Here, we produced eight single-domain fragments of Pfs230 in *Drosophila melanogaster* S2 cells and assessed their immunogenicity in mouse immunizations. In addition to D1-specific antibodies, antibodies raised against D12 showed strong functional transmission-reducing activity in membrane feeding assays with cultured parasites, an activity that was complement dependent. Murine D12-specific antibodies further reduced mosquito transmission of parasites acquired from naturally infected parasite carriers. The D12 antigen was recognized by sera from an all-age cohort of individuals who had been naturally exposed to *P. falciparum* with antibody levels increasing with age. In conclusion, we identified Pfs230D12 as a promising TBV candidate.

## Teaser

Pfs230D12 is a vaccine candidate inducing antibodies that prevent transmission of malaria parasites to mosquitoes.

## INTRODUCTION

The burden of malaria has increased in recent years, with 450,000 fatal malaria cases in 2016 rising to 608,000 in 2022 (*1*). Malaria is caused by *Plasmodium* parasites, of which *Plasmodium falciparum* is the deadliest, that are transmitted by *Anopheles* mosquitoes. Transmission to mosquitoes relies on the uptake of sexual stage parasites, female and male gametocytes, through a blood meal. Inside the mosquito midgut, gametocytes activate to become female macrogametes and exflagellating male microgametes that fertilize to form zygotes. From this point onward, *Plasmodium* parasites continue their life cycle by developing into ookinetes that traverse the midgut epithelium and form oocysts on the midgut basal side underneath the basal lamina. Within these oocysts, sporozoites that find their way to the salivary glands are formed, resulting in infectious mosquitoes. Transmission to mosquitoes forms a bottleneck in the life cycle of malaria parasites and is therefore an attractive target for interventions. Transmission-blocking vaccines (TBVs) target this bottleneck with the aim to reduce the number of mosquitoes that become infectious; TBVs thereby form valuable assets for malaria elimination strategies (*2*).

**Fig. 1.**
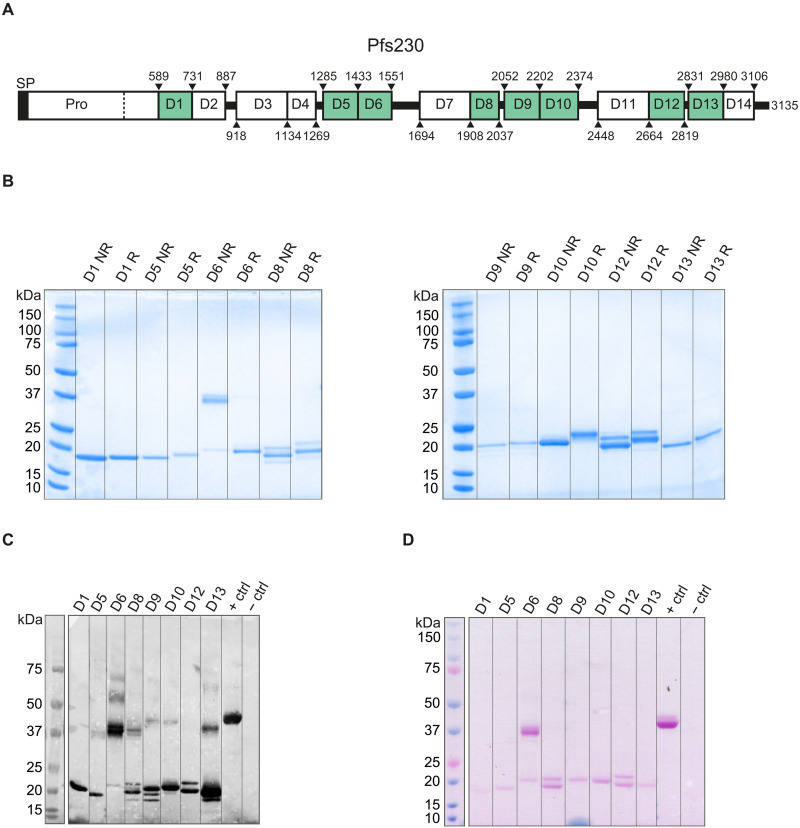
Recombinant single-domain Pfs230 proteins produced in *Drosophila melanogaster* S2 cells. (**A**) Overview of full-length Pfs230. Predicted domain boundaries are indicated by amino acid numbers and are based on predictions made by Gerloff *et al.* (*5*). Single-domain fragments with these boundaries were expressed in *D. melanogaster* S2 cells. Green coloring indicates successful expression of the fragment. (**B**) Coomassie-stained SDS-PAGE gel of purified single-domain fragments under reducing conditions (R) and nonreducing conditions (NR). (**C**) Western blot of recombinant fragments with α-C–tag antibody under nonreducing conditions. + and – controls are C-tagged protein and protein without C-tag, respectively. (**D**) Glycosylation-stained SDS-PAGE gel of recombinant fragments under nonreducing conditions. + and – controls are control proteins provided with the Pierce glycoprotein staining kit.

TBVs induce antibodies in the human host against surface antigens of gametes, zygotes, and/or ookinetes. These antibodies are taken up by the mosquito via the blood meal together with gametocytes and human complement. Inside the midgut, where parasites egress from the red blood cells (RBCs) and become accessible to antibodies; the antibodies prevent further development of the parasite through neutralization or activation of human complement that results in parasite lysis. The functional activity of malaria transmission–blocking antibodies is commonly quantified by measuring the reduction in oocyst numbers compared to a negative control and expressed as the percentage transmission-reducing activity (TRA). The clinically most advanced TBV candidate is Pfs230, which is essential for fertilization and further development into oocysts (*3*). Pfs230 is an abundant gamete surface protein that consists of fourteen 6-Cys domains (Fig. 1A) (*4*, *5*). The large size of Pfs230 hampers recombinant expression of full-length Pfs230, and vaccine development has focused on expression of fragments of the protein (*6*). Immunization studies found that the Pfs230 Pro-domain (Pro) and Domain 1 (D1) induced a functional response in rodents (*7*–*12*), which formed the basis for the development of Pfs230D1-EPA, the only TBV candidate to date that progressed to phase 2 clinical studies (*13*–*15*). For many years, it has been unclear whether domains outside Pro and D1 contain epitopes for functional antibodies. Recent studies showed that functional monoclonal antibodies (mAbs) induced by whole parasite immunization or natural exposure target Pfs230 epitopes outside ProD1 (*16*–*19*). However, recombinant fragments containing non-ProD1 fragments of Pfs230 have so far failed to induce a functional response in vivo (*7*–*9*), and ProD1 thus remains the only Pfs230-based vaccine candidate with demonstrated in vivo efficacy described to date.

Here, we expressed all single Pfs230 domains in *Drosophila melanogaster* S2 cells that have been successfully used for expression of the 6-Cys domain protein Pfs48/45 (*20*, *21*). We obtained eight pure single-domain fragments that were used to immunize mice. Of these fragments, D12 induced antibodies with strong TRA in membrane feeding assays with laboratory-cultured parasites and in membrane feeding assays with naturally circulating parasites from human donors. We also show that people with natural exposure to malaria parasites have antibodies that recognize Pfs230D12. These results position Pfs230D12 as a promising TBV candidate.

## RESULTS

### Production of single domain Pfs230 protein fragments

We expressed single-domain protein fragments with a C-terminal C-tag in *D. melanogaster* S2 cells (tables S1 and S2). Nine of the 14 constructs showed clear expression in S2 cell supernatants by Western blot with an α-C–tag antibody (fig. S1A). The cell lines that showed clear expression, i.e., lines expressing D1, D3, D5, D6, D8, D9, D10, D12, and D13, were scaled up, and proteins were purified using C-tag purification followed by size exclusion chromatography (Fig. 1B). Unlike the other domains, D3 and D9 showed strong aggregation by size exclusion chromatography. Using the mild detergent Empigen BB, we resolved aggregation for D9. D3 remained largely aggregated in the presence of Empigen BB and was excluded from further analyses.

We obtained pure D1, D5, D6, D8, D9, D10, D12 and D13 proteins as determined by SDS–polyacrylamide gel electrophoresis (SDS-PAGE) (Fig. 1B) and Western blot (Fig. 1C) analyses. The proteins showed a slight change in apparent mass between reducing and nonreducing conditions, indicating that they form intramolecular disulphide bonds, as can be expected for 6-Cys domain proteins (Fig. 1B, table S2). D6 appeared as a dimeric protein on SDS-PAGE, which was resolved by the addition of reducing agent, indicating intermolecular disulphide bond formation (Fig. 1B). All the antigens appear to be glycosylated, albeit to different extents (Fig. 1D). D12 ran as two different bands on SDS-PAGE gel. and both appeared to be glycosylated. Deglycosylation resulted in a single band of smaller size, demonstrating that the two bands represent two different glycoforms of D12 (fig. S1E). Together, we obtained eight Pfs230 single-domain antigens in sufficient quantity and purity for mouse immunizations.

### Pfs230D12 mouse antibodies recognize native Pfs230

**Fig. 2.**
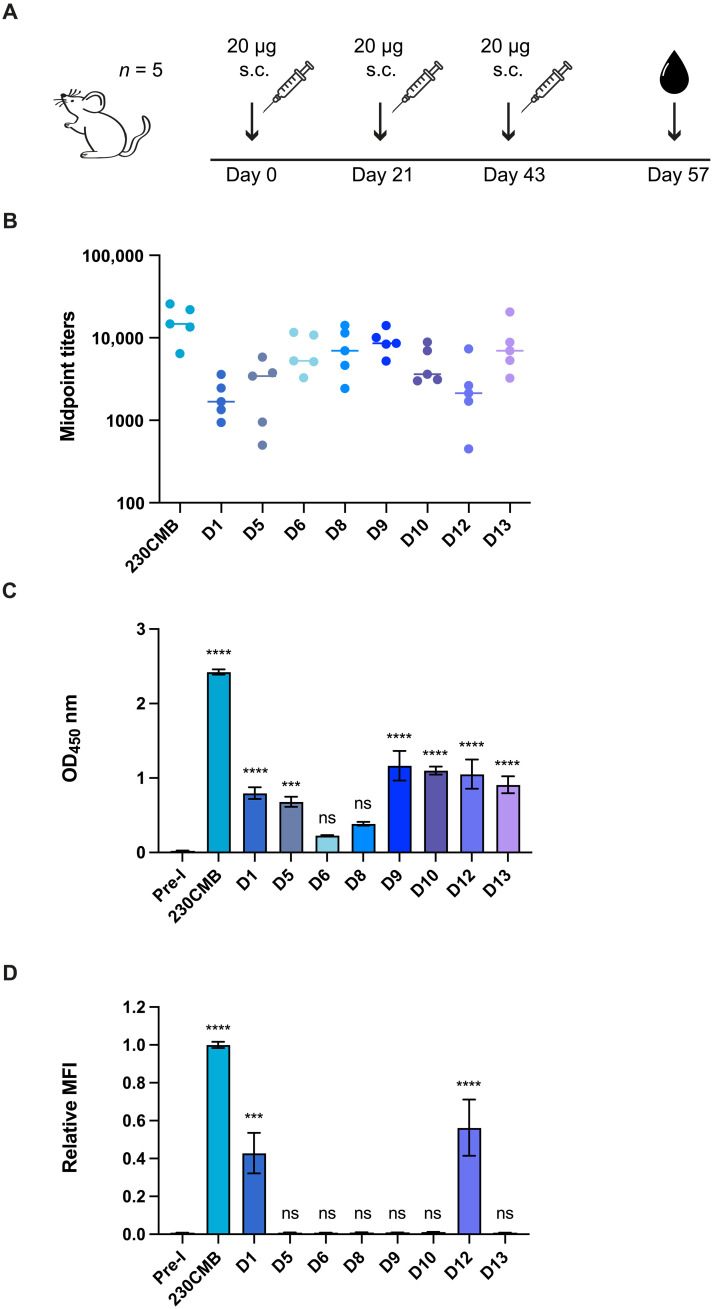
Antigen and parasite recognition by mouse antibodies raised against single Pfs230 domains. (**A**) Overview of mouse immunization regimen. Groups of five mice were immunized subcutaneously (s.c.) with 20 μg of antigen formulated in Montanide ISA-720. One group of mice was immunized with 230CMB (amino acids 444 to 730) (*21*) and was included as positive control. Final bleed sera were collected at day 57 for analyses of antibody responses in graphs (B) to (D). (**B**) Midpoint titers from antigen specific ELISA. Each dot represents an individual mouse, and bars represent median values. (**C**) Gametocyte extract ELISA with pooled mouse sera, tested at 1:100 dilution. Values are means from two independent experiments with three technical replicates each, and error bars represent SEM. Pre-I, preimmune serum. (**D**) Female gamete binding assay with pooled mouse sera, tested at 1:40 dilution. Values are means (MFI, mean fluorescence intensity) from two independent experiments with two technical replicates each, and error bars indicate SEM. The data were normalized against 230CMB in each independent experiment to allow averaging across experiments. Statistical analysis in (C) and (D) done by comparing test groups to preimmune group using ordinary one-way analysis of variance (ANOVA) with a Dunnett’s multiple comparison test using Pre-I as a reference (ns, not significant; ***P < 0.001; ****P < 0.0001).

For each selected Pfs230 protein construct, a group of five female mice was immunized three times with 20 μg of antigen formulated in Montanide ISA720 and blood was collected 14 days after the third immunization (Fig. 2A). A positive control group was immunized with 230CMB, a plant-produced protein containing the Pro and D1, which was previously shown to induce antibodies with strong TRA in rabbits (*22*). All mice generated antibody responses against the immunogen they were immunized with (Fig. 2B and fig. S2). One mouse in the D12 group showed very low antibody responses (fig. S2), and sera from this mouse were therefore excluded from further analyses. Antibodies in pooled mouse sera recognized native Pfs230 in enzyme-linked immunosorbent assays (ELISA) with gametocyte extract at different intensities (Fig. 2C). Sera raised against D1, D5, D9, D10, D12, and D13 showed statistically significantly higher recognition compared to preimmune sera. Recognition by sera against D6 and D8 was weaker and not statistically significant. We also tested recognition of native Pfs230 on the surface of live female gametes. Strikingly, only sera generated against 230CMB, D1, and D12 bound to the surface of female gametes as assessed by flow cytometry (Fig. 2D) and microscopy (fig. S3). Together, the results indicate that while sera raised against most single-domain constructs recognize Pfs230 in parasite extract, only sera against D1 and D12 recognize Pfs230 on live female gametes.

### Pfs230D12 antibodies block transmission of *P. falciparum* NF54

**Fig. 3.**
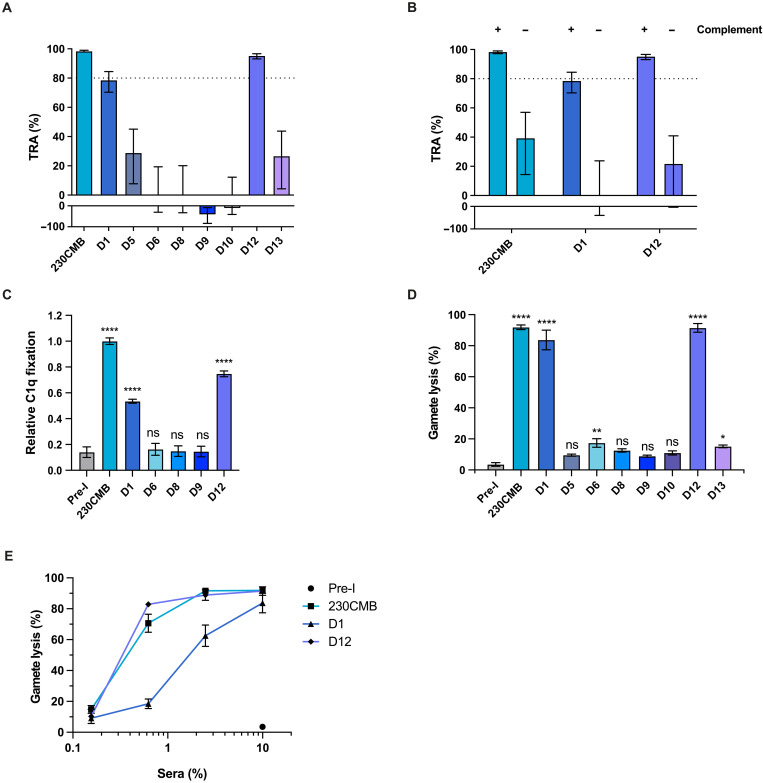
Functional activity of mouse antibodies against single Pfs230 domains. (**A**) TRA of pooled mouse sera (day 57) in SMFA with cultured *Plasmodium falciparum* NF54 gametocytes and *Anopheles stephensi* mosquitoes. Values are estimates from two independent SMFA experiments with oocyst counts for 16 to 20 fully fed mosquitoes per condition each. Dotted line indicates 80% TRA, which has previously been established as threshold for clinical development (*22*). Error bars indicate 95% CIs. Sera were tested at a dilution of 1:9 in the presence of active human complement. (**B**) TRA of pooled mouse sera in SMFA, in the presence of active (+) or heat-inactivated (−) human complement. Pooled mouse sera were tested at a final dilution of 1:9. Values are estimates from two independent experiments with oocysts counts for 20 fully fed mosquitoes per condition per experiment. (**C**) C1q deposition on the surface of female gametes in the presence of 2.5% pooled mouse serum, as assessed by flow cytometry. Values are means from two independent experiments with two technical replicates each, and error bars indicate SEM. Data were normalized against 230CMB to allow averaging across experiments. Pre-I, preimmune serum. (**D**) Female gamete lysis assay with 10% pooled mouse serum and active human complement. Values are means from two independent experiments with two technical replicates each, and error bars indicate SEM. 0% lysis is defined as the number of live gametes after incubation with human complement only. Statistical analysis in (C) and (D) was done by comparing test groups to pre-immune group using ordinary one-way ANOVA and accounted for multiple comparisons by Dunnett’s multiple comparison test (ns, not significant; *P < 0.05; **P < 0.01; ****P < 0.0001). (**E**) Pooled mouse sera were titrated in the gamete lysis assay. Values are means from two independent experiments with two technical replicates each, and error bars indicate SEM.

To assess functional activity of the domain-specific antibodies, we performed standard membrane feeding assays (SMFAs). In these assays, we allowed laboratory-reared *Anopheles stephensi* mosquitoes to feed on a mixture of cultured *P. falciparum* NF54 gametocytes and sera from immunized mice, and after 6 to 8 days, we counted oocysts in the mosquito midgut to calculate TRA. In line with previous studies, sera raised against 230CMB and D1 showed strong TRA when tested at ninefold dilution, reducing oocyst formation by 98.3% [95% confidence intervals (CIs): 97.2 to 99.0] and 78.5% (95% CIs: 70.3 to 84.4), respectively (Fig. 3A). D12 sera also showed strong TRA (95.2% TRA, 95% CIs: 93.1 to 96.6). Testing more dilute 230CMB and D12 sera showed that these sera retained TRAs of >80% at 72-fold and 36-fold dilution, respectively (fig. S4). At 36-fold dilution, D12 sera showed a strong TRAs of 92.1% (95% CIs: 89.6 to 94.0). Sera raised against other domains showed very low or no TRA, which is consistent with the binding assays where the antibodies in these sera failed to recognize the gamete surface (Fig. 2D). Mass spectrometry confirmed purity of the D12 immunogen (tables S3 and S4), and Western blots with gametocyte extract and single-domain fragments expressed with the wheat germ cell–free system further confirmed that the functional antibodies were D12-specific (fig. S5).

Since the vast majority of functional Pfs230 antibodies described to date are complement dependent, we tested sera against 230CMB, D1, and D12 in SMFA with either active or heat-inactivated human complement (Fig. 3B). Sera showed substantial TRA only in the presence of active complement, demonstrating the complement dependency of antibodies against these domains. To confirm that the complement-dependent activity is mediated by classical pathway activation, the ability of the mouse antibodies to fix C1q on the parasite surface was assessed in a flow cytometry assay with live female gametes. Antibodies against 230CMB, D1, and D12 were able to mediate C1q deposition, while antibodies against D6, D8, and D9 failed to do so (Fig. 3C), in line with the SMFA results. We then tested whether the deposition of C1q leads to lysis of the female gamete in a flow cytometry lysis assay (Fig. 3, D and E). In this assay, purified female gametes are incubated with mouse sera and human complement, and, after incubation, the percentage lysis is determined by live/dead staining. Sera against 230CMB, D1, and D12 showed more than 80% lysis when tested at 1:10 dilution (Fig. 3D). Other sera showed little, in most cases nonsignificant, lysis, which is consistent with results from the C1q deposition assay and SMFA. Titration of mice sera demonstrated similar potency for 230CMB and D12 sera, while D1 sera had slightly lower potency, similar to the trend observed in SMFA. Together, these data indicate that the D12 antigen can induce functional antibodies in mice capable of reducing transmission of laboratory-cultured malaria parasites to mosquitoes in a complement-dependent manner.

### Murine Pfs230D12 antibodies reduce transmission of naturally circulating gametocytes

**Fig. 4.**
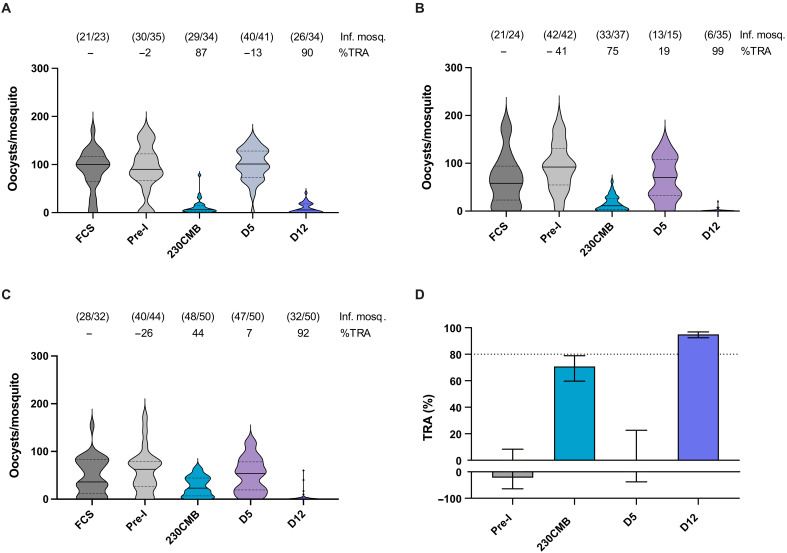
TRA of Pfs230D12 sera in DMFA with naturally circulating gametocyte strains from volunteers in Burkina Faso. (**A** to **C**) Gametocytes from three volunteers were fed to *Anopheles coluzzii* mosquitoes in the presence of mouse sera (day 57) and human complement. Pre-I, pre-immune serum. Data are shown as violin plots with the median indicated as a line and the interquartile range (IQR) (Q1 and Q3 quartiles) as dotted lines. Values above bars indicate percentage TRA and the number of infected mosquitoes and the total number of mosquitoes between brackets (# infected mosquitoes/# total mosquitoes). (**D**) Estimate TRA values from three independent experiments (A to C) combined. Bars are estimated means, and error bars indicate 95% CIs. Dotted line indicates 80% TRA, which has previously been established as threshold for clinical development (*22*).

To assess the functionality of D12 antibodies against naturally circulating gametocyte strains, we performed direct membrane feeding assays (DMFA) using blood of naturally infected gametocyte carriers from Burkina Faso. After removal of autologous plasma, RBCs were mixed with pooled mouse sera and normal human serum (NHS) containing complement and fed to mosquitoes. After 7 days, oocysts were counted and TRA was calculated. We tested 230CMB and D12 sera that both showed strong TRA in SMFA and further included preimmune and D5 sera as negative controls. While D5 sera did not reduce oocyst formation, in line with SMFA results, the 230CMB and D12 sera reduced oocyst numbers across three independent experiments (Fig. 4, A to C). The estimated TRA values were 70.8% (95% CIs: 59.7 to 78.9) and 95.1% (95% CIs: 92.4 to 96.8) for 230CMB and D12 sera, respectively (Fig. 4D). The DMFA results thus show that D12 antibodies have strong TRA against naturally circulating gametocytes.

### Pfs230D12 is recognized by sera from individuals naturally exposed to *P. falciparum*

**Fig. 5.**
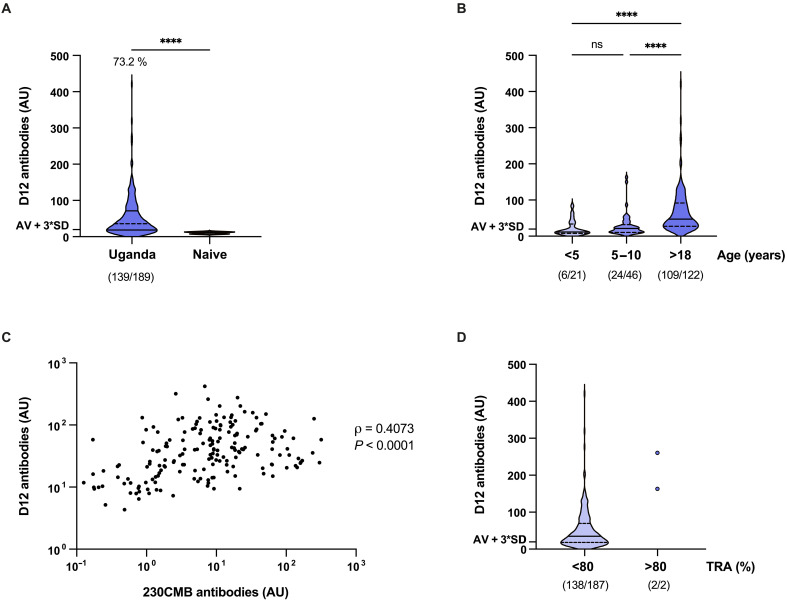
Recognition of Pfs230D12 by plasma from volunteers naturally exposed to *P. falciparum*. (**A**) Antibody levels against D12 in Ugandan plasma samples, as determined by ELISA. AU are arbitrary units calculated using a highly reactive plasma pool from Tanzania as reference. Naïve samples are pooled plasma samples from malaria-naïve Dutch donors (*n* = 8). Groups were compared by one-sided Mann-Whitney test (****P < 0.0001). The threshold for positivity is marked and defined as the mean of naïve controls plus three SDs (AV + 3*SD). Percentage of positive samples is indicated above the graph. The number of positive samples and the total number of samples are depicted in between brackets (# positive samples/# total samples). (**B**) Antibody levels stratified by age. Groups were compared by Kruskal-Wallis test with Dunn’s correction for multiple testing (ns, not significant; ****P < 0.0001). The number of positive samples and the total number of samples per age group are depicted in between brackets (#positive samples/# total samples). (**C**) Correlation between antibody levels against D12 and 230CMB (Spearman’s ρ = 0.4073, *P* < 0.0001). Arbitrary units (AU) against 230CMB are calculated using serum from an individual that had high levels of antibodies against D12 [donor A (*24*)]. Five individuals with 230CMB-specific antibody levels below 0.1 AU are not shown on graph. (**D**) Antibody levels stratified by TRA. Number of antibody positive over total tested individuals per group are shown below the graph. Data in (A), (B), and (D) are shown as violin plots with the median indicated as a line and the IQR (Q1 and Q3 quartiles) as dotted lines.

To assess natural antibody responses to D12, we screened plasma samples from a cohort of individuals residing in Tororo, an area in eastern Uganda where, at the time of sampling (2013 to 2017), transmission intensity was intense and perennial (*23*). Children ≤10 years of age and adults were eligible for enrolment. Purified immunoglobulin G (IgG) samples of these cohort participants were tested for transmission-reducing immune responses in the SMFA, as described above, as part of a larger study on naturally acquired transmission-reducing immunity. We detected significantly higher antibody levels in Ugandan samples compared to naïve control samples from Dutch donors (Mann-Whitney test, *P* < 0.0001) (Fig. 5A). A total of 139 of 189 (73.2%) individuals were seropositive for D12. Antibody intensity was significantly higher in adults compared to school-aged children (5 to 10 years old) and younger children (<5 years old) (Kruskal-Wallis test, *P* < 0.0001) (Fig. 5B). We observed a statistically significant correlation between antibody responses against 230CMB and D12 (Spearman’s, ρ = 0.4073, *P* < 0.0001) (Fig. 5C). While numbers were too small for meaningful statistical comparisons, we observed very strong D12 responses in two individuals whose total IgG isolated from plasma showed strong TRA in SMFA (Fig. 5D). Together, we found that individuals living in a malaria-endemic country can generate antibodies against D12 and that antibody levels increase with age.

## DISCUSSION

Here, we expressed eight individual domains of Pfs230, a *P. falciparum* protein that is essential for parasite transmission and forms the basis of current malaria TBV development. Antibodies raised against the D12 domain showed potent binding to live female gametes in vitro and have strong functional TRA. Functional activity was demonstrated against laboratory-cultured gametocytes and gametocytes from naturally infected parasite carriers from Burkina Faso. Furthermore, sera from Ugandan donors naturally exposed to *P. falciparum* showed immune recognition of D12 in an age-dependent manner. Together, our results identify Pfs230D12 as a promising TBV candidate.

TBVs could be valuable tools for the elimination and eradication of malaria. Several TBV candidates have been identified, of which the ProD1 fragment of Pfs230 has progressed furthest in terms of clinical testing. This study aimed to comprehensively examine constructs outside ProD1 and found that D12 can elicit functional antibodies. Two earlier immunization studies included fragments containing D12, produced in *Escherichia coli* (*8*) and wheat germ cell–free system (*9*). These constructs induced antibodies that recognized native Pfs230 on gametes, but the antibodies did not reduce transmission to mosquitoes (*8*, *9*). This is in sharp contrast with our results that show high TRA for mouse antibodies raised against D12 produced in *D. melanogaster* S2 cells. It is likely that the expression system plays an important role with *D. melanogaster* S2 cells producing (more) properly folded D12 that is critical for raising functional antibodies (Fig. 1B). This is in line with preclinical studies with the other 6-Cys family protein Pfs48/45 that showed that the host expression system and proper conformation of the antigen are essential for inducing functional responses (*24*).

While antibodies raised against the D12 antigen showed strong gamete recognition and functional TRA, we did not observe functional responses against D5, D6, D8, D9, D10, and D13 (Fig. 3A). Strikingly, antibodies against most of these domains recognized native Pfs230 in gametocyte extract but did not recognize Pfs230 on live gametes (Fig. 2, C and D), suggesting that epitopes on these domains are occluded by other parasite surface proteins that interact with, or are in close proximity of, Pfs230. Alternatively, epitopes on these domains may be close to the parasite membrane and therefore not accessible to antibodies. However, we cannot rule out that these domains of Pfs230 do contain functional epitopes, as the functional epitopes could have been absent in our recombinant antigens due to misfolding or masking by glycosylation in the *D. melanogaster* S2 cell expression system (Fig. 1C). Recently emerged cryogenic electron microscopy structures of the native full-length Pfs48/45:Pfs230 complex provide further insight into which Pfs230 domains are membrane distal and surface exposed, and, hence, more likely to contain functional epitopes (*25*, *26*).

Most if not all functional Pfs230 antibodies described to date are dependent on complement (*4*, *12*, *13*, *16*, *27*–*29*). The antibodies we raised against D12 are no exception to this rule. The complement dependency of the D12 antibodies was shown in membrane feeding assays where antibodies lacked TRA in the presence of heat-inactivated complement (Fig. 3B). The D12 antibodies can activate the classical pathway by fixing C1q (Fig. 3C) and induce complement-mediated gamete lysis in vitro (Fig. 3, D and E), which is the presumed effector mechanism in the mosquito midgut.

The viability of D12 as vaccine candidate depends on several factors. In general, vaccine candidates should target conserved functional epitopes to generate cross-strain protection, should ideally be able to induce highly potent antibodies so lower overall antibody responses are needed for protection, and should be immunogenic in humans. Like other sexual stage *Plasmodium* proteins, Pfs230 is well conserved; D12 contains a similar number of nonsynonymous single-nucleotide polymorphisms, approximately 10-per-kb coding sequence, as the leading TBV candidate ProD1 (*17*). The D12-specific mouse antibodies not only block transmission of the reference strain *P. falciparum* NF54 but also block transmission of naturally circulating gametocytes that may be genetically diverse (Fig. 4). However, whether the functional antibodies indeed target conserved epitopes on D12 is currently unknown and should be the focus of future research. This could be addressed by isolating and characterizing mAbs , either from immunized animals or from human donors with naturally acquired immunity, and assessing their structure-function relationships as recently done for ProD1 (*27*, *28*, *30*). Furthermore, we observed that many individuals in a Ugandan cohort were seropositive for D12, demonstrating that the D12 antigen is immunogenic in humans and that vaccine-induced antibody levels may be boosted by natural exposure and vice versa. Naturally acquired transmission reducing immunity is a rare phenomenon, at least at high levels of TRA that can be reproducibly demonstrated in the SMFA (*31*). While now allowing for a formal assessment of a possible role of D12-specific antibodies in naturally acquired TRA, we made use of a larger cohort study where plasma samples were available alongside TRA estimates. Two individuals with high levels of TRA also had high levels of antibodies against D12. The strong correlation between anti-D12 antibodies and anti-Pfs230CMB antibodies makes it impossible to determine whether D12 antibodies were causally responsible for TRA. Purification of D12-specific antibodies from plasma and assessment of their TRA in SMFA (*31*) would allow us to demonstrate causality, but this was not possible with the plasma volumes available. Nevertheless, our findings demonstrate that D12 antibodies are naturally acquired in an age-dependent manner—probably reflecting cumulative exposure to *P. falciparum* gametocytes—in a manner that is similar to antibodies to other Pfs230 domains and that individuals with naturally acquired TRA can have high levels of D12 antibodies.

In our immunization studies, we included 230CMB (*22*), containing ProD1, as positive control. It is encouraging to see that our nonoptimized D12 immunogen induced TRA levels that are close to those induced by 230CMB (Fig. 3A, Fig. 4C, and fig. S4) and that D12 sera showed similar gamete lysis activity as ProD1 sera (Fig. 3E). Future studies should assess whether the D12 antigen could be further optimized to induce stronger functional responses, for instance, through coupling to carrier proteins, screening of glycosylation mutants, adjuvant screening, and/or designing immunogen variants that are more stable or have improved epitope display. It will also be interesting to determine whether antibodies against D12 are synergistic or additive with antibodies against other TBV candidates such as Pfs230D1-EPA, as combining antigens may be an attractive approach to increase overall vaccine efficacy, and to formally compare the potency of different vaccine candidates.

This study has several limitations. We aimed to assess all single domains of Pfs230 in our immunization study but could not produce 6 of the 14 Pfs230 domains. These included D4 and D7, which have been shown to be targets for functional mAbs raised against whole parasites (*17*, *18*). Whether these domains can be produced recombinantly to induce functional antibodies thus remains unclear. Furthermore, one of the mice in the D12 group showed very low antibody responses to the immunogen (fig. S2). Whether this low response is linked to the immunogen itself or whether this was caused by external factors is currently unclear and should be assessed in future immunization studies. Last, we could test the mouse sera in only three DMFA experiments due to logistical challenges. The results from these DMFA experiments should therefore be interpreted with caution and warrant more in-depth studies to assess cross-strain protection. In conclusion, our work shows how using a different expression system to produce recombinant domains of Pfs230 can lead to the identification of a malaria TBV candidate and that Pfs230D12 is a promising candidate for further preclinical investigation.

## MATERIALS AND METHODS

### Protein construct design

Expression plasmids were created for all 14 domains of Pfs230 (Fig. 1A). Boundaries of the domains were based on previous research (*5*), and the linkers between domains were excluded. Sequences were codon optimized for expression in *D. melanogaster* and synthesized (BaseClear). The Pfs230 single domains were cloned with an N-terminal BiP signal peptide, His_6_-tag, and alanine-serine linker, and a C-terminal glycine-serine linker followed by a C-tag into the pExpreS2.2 plasmid (ExpreS2ion Biotechnologies), downstream of the Actin+HSP70 promoter. The plasmids were verified by Sanger sequencing (Baseclear). The sequences of the inserts can be found in table S1.

### *D. melanogaster* S2 cell transfection and culture

The *D. melanogaster* S2 cell line (ExpreS2ion Biotechnologies) was used for the expression of all Pfs230 protein constructs. S2 cells were cultured in shake flasks with vented cap in EX-CELL420 media (Sigma-Aldrich), supplemented with 1% penicillin-streptomycin, at 25°C shaking 115 rpm. Cells were counted twice a week and resuspended to 8 × 10^6^ cells/ml alternately by dilution or centrifugation. For transfections, 2.5 ml of cell suspension was mixed with 6.25 μg of plasmid DNA and 25 μl of ExpreS2 Insect-TR 5x transfection reagent (ExpreS2ion Biotechnologies) in a T12.5 T-flask. The transfected cells were then incubated at 25°C, and 1 ml of fetal bovine serum (FBS) was added after 3 hours. Geneticin (4000 μg/ml) was added as a selection agent after 24 hours. Approximately 26 days after the transfection, the cultures were scaled up to shake flasks. During this step, FBS and geneticin were removed by centrifugation and resuspending the cells in EX-CELL420 to 8 × 10^6^ cells/ml. Supernatant was harvested 5 days after the cells were diluted for protein expression analysis on Western blot and protein purification.

### Protein purification

The S2 cell supernatant was concentrated from 200 to 300 ml to approximately 50 ml using the Masterflex EasyLoad (Masterflex). The Pfs230 single-domain protein constructs were affinity purified with CaptureSelect C-tagXL prepacked columns (Thermo Fisher Scientific) on an ÄKTA start (Cytiva) using 20 mM tris wash buffer (pH 7.4) and 20 mM tris + 2 M MgCl_2_ (pH 7.4) elution buffer. Peak fractions from the chromatogram were pooled and dialyzed overnight in phosphate-buffered saline (PBS). The sample was filtered and concentrated to approximately 600 μl. Subsequently, the sample was further purified using a Superdex75 10/300 GL column (Cytiva) with filtered and degassed PBS as running buffer. For Pfs230D9 and Pfs230D3 0.2% Empigen BB (Sigma-Aldrich) was added to all purification buffers to decrease aggregation of the proteins. Superdex fractions containing pure monomer protein were pooled and the protein concentration measured using a Nanodrop spectrophotometer (Thermo Fisher Scientific). Samples were frozen in liquid nitrogen and stored at −70°C.

### SDS-PAGE analysis

For analysis of proteins from S2 expression, samples were mixed with 4× NuPAGE LDS sample buffer (Invitrogen), heated at 70°C for 10 min before loading on a 4 to 20% bis-tris polyacrylamide gel (GenScript). In the case of purified protein, 1 μg of protein was loaded per condition and the Precision Plus Dual Color protein marker (Bio-Rad) was used as size standard. The gels were stained for 30 min using Instant Blue Coomassie Protein Stain (Abcam). To reduce disulphide bonds, a final concentration of 10 mM dithiothreitol (DDT) was added in the preparation of the sample.

For protein analysis with parasite extract, *P. falciparum* NF54 gametocyte extract was prepared as described previously (*32*) and diluted to the equivalent of 500,000 gametocytes per well. A final concentration of 10 mM DTT was added for reducing conditions. Gametocyte extract and 230CMB (*22*) samples were mixed with 4× NuPAGE LDS sample buffer and heated for 10 min at 70°C before loading on a 4 to 20% bis-tris gel (GenScript). Twenty nanograms of 230CMB was loaded per well, and the Precision Plus Dual Color protein marker (Bio-Rad) was used as size standard.

For analysis of proteins expressed in wheat germ cell–free system, purified proteins [described previously (*9*, *18*)] were mixed with SDS-sample buffer and TCEP-HCl (Pierce) and denatured at 37°C for 30 min before loading on a 12.5% PAGE tris gel (ATTO, Tokyo, Japan). A total of 0.5 μg of each protein was loaded per well, and Precision Plus Protein All blue standard (Bio-rad) was used as the size standard.

### Western blot analysis

For Western blot analysis of proteins expressed in S2 cells, a positive control with C-tag (Pro-CS3-6C, gifted by S. Singh, 36.3 kDa) and negative control (Pf3D7_1306500C no C-tag, transfected in S2 cells) was included. The bis-tris gels were blotted on a 0.45-μm nitrocellulose membrane using the TurboBlot system (Bio-Rad). The membranes were washed in between steps with PBS supplemented with 0.05% Tween20 (PBST), blocked overnight in 5% skimmed milk PBS (mPBS) at 4°C, and incubated for 1 hour at room temperature (RT) with the CaptureSelect Biotin Anti-C-tag conjugate (1/1000, catalog no. 7103252100, Thermo Fisher Scientific) in 1% mPBST. Thereafter the membranes were incubated with 1/2500 IRDye Streptavidin 680LT (catalog no. 926-68031, LI-COR) in 1% mPBST for 1 hour at RT. The blot was developed with Clarity Max Western ECL substrate (Bio-Rad) and imaged with the Odyssey CLX (LI-COR).

For Western blots with gametocyte extract, gels were transferred to a 0.45-μm nitrocellulose membrane using the Trans-Blot Turbo transfer system (Bio-Rad). The blots were blocked with 5% skimmed milk in PBS before incubation with 1/5000 polyclonal serum from mice immunized with Pfs230D12. After washing, the strips were incubated with 1/3000 diluted polyclonal rabbit anti-mouse IgG horseradish peroxidase (HRP, catalog no. P0260, DAKO). Blots were developed with Clarity Max Western ECL substrate (Bio-Rad) and imaged on the ImageQuant LAS 4000 (GE Healthcare).

For Western blot analysis of proteins expressed in wheat germ cell–free system, the SDS-PAGE gels were transferred to an Amersham Hybond P low-fluorescence 0.2-μm polyvinylidene difluoride membrane (Cytiva) using the Trans-blot SD semidry transfer cell (Bio-Rad) (25 V, 126 mA per gel, 75 min). The blots were blocked with 5% skimmed milk in PBST before incubation with 1/1000 polyclonal serum from mice immunized with Pfs230D12 (diluted in PBST) for 1 hour at RT and overnight at 4°C. After washing, the blots were incubated with 1/10,000 polyclonal sheep anti-mouse IgG HRP (catalog no. NA931VS, Cytiva). Blots were developed with Immobilon Western chemiluminescent HRP substrate (catalog no. WBKLS0500, Millipore) and imaged on the LAS-4000 (FUJIFILM, Tokyo, Japan) for 13 min.

### Glycosylation staining and deglycosylation

Glycosylation of Pfs230 domains was assessed using the Pierce Glycoprotein Staining Kit (Thermo Fisher Scientific). Five micrograms of each Pfs230 protein construct was loaded on gel, and the gel was stained following the manufacturer’s instructions. D12 was deglycosylated with PNGase F (New England Biolabs) under denaturing/reducing conditions, following the manufacturer’s protocol. D12 was denatured with 0.5% SDS and 40 mM DTT for 10 min at 100°C. After cooling down the sample on ice, 1% NP-40, 50 mM sodium phosphate (pH 7.5) and PNGase F were added and the sample was incubated for 1 hour at 37°C, followed by analysis on SDS-PAGE gel.

### Mass spectrometry

The identity of recombinant Pfs230D12 was confirmed by mass spectrometry. Five micrograms of purified protein was denatured in 4 M urea, 100 mM tris-HCl (pH 8.0), and disulfide bonds were reduced using 10 mM DTT for 30 min at RT. Cysteines were alkylated using 50 mM iodoacetamide for 30 min, and samples were diluted to 2 M urea, using 100 mM tris-HCl (pH 8.0). Samples were digested overnight with 0.5 μg trypsin at 25°C. Next day, samples were desalted using StageTips (*33*).

Peptides were analyzed using an Easy nLC 1000 equiped with a 30-cm reverse phase column, coupled on-line to an Orbitrap Fusion Tribrid mass spectrometer (Thermo Fisher Scientific). A 60-min gradient of buffer B (80% acetonitrile and 0.1% formic acid) was applied, and the mass spectrometer was operated in TopS mode with a dynamic exclusion of 60 s.

RAW data were analyzed using Maxquant (*34*) version 1.6.6.0 with a *Drosophila* database supplemented with sequences for single-domain constructs of Pfs230. The mass spectrometry proteomics data have been deposited to the ProteomeXchange Consortium via the PRIDE partner repository with the dataset identifier PXD039716 (*35*).

### Mice immunization

Forty-five female 6- to 8-week-old CD-1 mice (Charles Rivers), divided in groups of 5 mice, were immunized with 230CMB, D1, D5, D6, D8, D9, D10, D12, and D13. 230CMB, a construct comprising amino acids 444 to 730 of Pfs230 produced in a plant-based expression system (*22*) and known to induce transmission reducing capacity was included as positive control. Mice were injected subcutaneously with 100 μl of antigen (0.2 mg/ml) in 70% Montanide ISA720 (SEPPIC) at day 0, day 21, and day 43. Prebleed samples were collected at day −1, and final bleeding was performed at day 57 before euthanasia (Fig. 2A). Blood was allowed to clot at RT for 30 min, and serum was collected after centrifugation and was stored at −20°C. For each group of mice sera, samples were pooled for further analysis. All animal procedures complied with national regulations and were approved by the ethics committee of the Radboud University Medical Center.

### Enzyme-linked immunosorbent assays

For antigen ELISA to assess antigen-specific antibody responses in mice, Nunc MaxoSorp 96-well plates (Thermo Fisher Scientific) were coated with 100 μl of antigen (1 μg/ml and incubated overnight at 4°C. Plates were washed three times with PBS in between incubation steps. Plates were blocked with 5% mPBS for 1 hour. Plates were incubated with serum samples diluted in 1% mPBST for 3 hours at RT. Subsequently, the plates were incubated with polyclonal rabbit anti-mouse HRP (1/3000 dilution, catalog no. P0260, DAKO) for 2 hours at RT. The ELISA was developed by adding 100 μl of tetramethylbenzidine. The color reaction was stopped by adding 50 μl of 0.2 M H_2_SO_4_ and the optical density was read at 450 nm on an iMark microplate absorbance reader (Bio-Rad).

For the gametocyte ELISA, *P. falciparum* NF54 gametocyte extracts were prepared as described previously (*32*). One hundred microliters of lysate per well, equivalent to 75,000 gametocytes, was pipetted into Nunc MaxiSorp 96-well plates (Thermo Fisher Scientific) and incubated overnight at 4°C. The next steps of the ELISA were performed as described above.

D12-specific antibody levels in sera from individuals exposed to malaria parasites were assessed using an antigen ELISA as described above. Goat anti-human IgG (H + L) HRP (1/40,000 dilution, catalog no. 31412, Invitrogen) was used for detection. A titration of pooled hyperimmune serum from gametocyte carriers in Tanzania was used to calculate arbitrary units using ADAMSEL FPL (http://www.malariaresearch.eu/content/software).

### Gamete purification

To obtain purified female gametes for flow cytometry assays, we collected *N*-acetyl glucosamine–treated 16-day-old *P. falciparum* NF54 gametocyte cultures. The cultures were centrifuged for 10 min at 2000*g* at RT to be resuspended in FBS using a volume that equals half the original culture volume. Gametocytes were placed on a roller bank for 45 min at RT for activation and thereafter centrifuged for 10 min at 2000*g* at 4°C. The pelleted gametes were resuspended in 1 ml of PBS, loaded onto a 7-ml layer of 11% w/v Accudenz (Accurate Chemical), and centrifuged for 30 min at 7000*g* at 4°C without brake (Sorvall RC-5B Superspeed Centrifuge with HB-4 swing-out rotor). The female gametes present in the top layer were collected, transferred to a 50-ml tube, and PBS was added up to 50 ml of total volume. A final centrifugation for 5 min at 2000*g* at 4°C was done to pellet the gametes, which were resuspended in 1 ml of PBS and counted using a Bürker-Turk counting chamber.

### Flow cytometry

For the assessment of gamete antibody binding, gamete C1q deposition, and gamete lysis, we used similar flow cytometry assays with specific adjustments that are described in this paragraph. All gamete incubations were carried out in PBS supplemented with 2% FBS and 0.02% sodium azide. For all three assays, 50,000 purified gametes were used per well in a V-bottom nontreated 96-well plate (Costar) and were incubated for 1 hour at RT with mice sera. In the case of a lysis assay, there is an addition of 20% NHS and the incubation is reduced to 30 min at RT. For a C1q deposition assay, this is reduced to 10% NHS (30 min of incubation at RT). Plates were centrifuged at 2000*g* for 3 min at 4°C and washed three times with PBS. The C1q deposition assay includes additional steps; First, PBS supplemented with 10 mM EDTA is added for 5 min at 4°C to inactivate complement. Second, after three washes with PBS, 1/5000 anti-C1q goat anti-human polyclonal serum (Complement Technology) is added for 30 min incubation at RT. Gametes were washed and then incubated with either 1/200 Alexa Fluor 488 chicken anti-mouse IgG (H + L) (Invitrogen) (binding assay), 1/200 anti-Pfs47 (rat mAb 47.1) (*36*) labeled with DyLight 650 NHS ester (Thermo Fisher Scientific) (lysis assay) or 1/200 Alexa Fluor 488 donkey anti-goat IgG (H + L) (Invitrogen). eBioscience Fixable Viability Dye eFluor 780 (1/1000, Invitrogen) was added in all assays, and gametes were incubated for 30 min at RT. After washing with PBS, the samples were resuspended in 150 μl of PBS. Antibody binding to gametes, lysis of gametes, and C1q deposition on gametes were assessed by flow cytometry by analyzing a minimum of 2000 gametes with the Gallios 10-color system (Beckman Coulter) and analyzed with FlowJo (BD, version 10.7.1) (gating strategy in fig. S6).

### Surface immunofluorescence assay

Fourteen-day-old heparin-treated *P. falciparum* NF54 gametocyte cultures were spun down, reconstituted in half the volume fetal calf serum (FCS) and incubated for 1 hour at RT to generate female gametes. Gametes were washed with SIFA buffer (0.5% FCS in PBS) three times. Cells containing female gametes were incubated with pooled mouse serum diluted in SIFA buffer for 1 hour at 4°C. Cells were washed three times with SIFA buffer, after which they were incubated with 1:200 diluted Alexa Fluor 488 goat anti-mouse IgG (H + L) (Invitrogen, A11029) for 1 hour at 4°C. Cells were washed again with SIFA buffer and imaged using an Axio Observer 7 Inverted light-emitting diode (LED) microscope equipped with a Colibri 7 LED source and Axiocam 705 mono (Zeiss). Female gametes were first searched in the bright-field channel, after which fluorescence was assessed using the 475-nm LED module.

### Standard membrane feeding assay

Mice sera were diluted in FCS to a final volume of 90 μl and mixed with 30 μl of NHS and 180 μl of mature *P. falciparum* NF54 gametocytes and RBCs. Indicated serum dilutions are calculated relative to the final blood meal volume, e.g., a mouse sample tested at a ninefold dilution contains 30 μl of mouse serum in a total blood meal volume of 270 μl. To inactivate NHS for conditions where inactive complement is required, it was heated for 30 min at 56°C before mixing with the gametocytes. *A. stephensi* mosquitoes from a colony maintained at Radboudumc (Nijmegen, the Netherlands) were fed blood meals as described previously (*37*). Unfed and partially fed mosquitoes were removed. Twenty mosquitoes per condition were dissected 6 to 8 days after the blood meal to collect their midguts. The midguts were stained with mercurochrome, and oocysts were counted. TRA was defined as the reduction in oocyst intensity (oocysts per mosquito midgut) in a test condition compared to a negative control in which no mice sera (FCS control) was added. All samples were tested in two independent SMFA experiments for which the oocyst count data are shown in data file S1.

### Direct membrane feeding assay

Gametocyte-infected blood from patients residing in the villages surrounding Bobo-Dioulasso was collected in heparin tubes, 5 ml per tube. Immediately after blood collection, the blood was centrifuged at 3000*g* for 5 min, and plasma was removed. One hundred and twenty microliters of the remaining RBC pellet was transferred to tubes containing 90 μl of naïve AB serum and 30 μl of mice sera (or FCS as negative control). The total of 240 μl was carefully mixed by pipetting, and the content of each tube was transferred to an individual feeder maintained at 37°C to allow *Anopheles coluzzii* mosquito feeding for 30 min. Unfed mosquitoes were removed; fullyfed mosquitoes were kept for 7 days postfeeding. All surviving mosquitoes were dissected for each condition, midguts were stained with 0.5% mercurochrome for oocyst detection, and oocysts were counted.

### Ethics statement

Asymptomatic gametocyte carriers, aged 7 and 10 years, were enrolled in November 2023 from the villages surrounding Bobo Dioulasso (Burkina Faso). Venous blood samples of the two volunteers were collected after written informed consent was obtained from participants or their guardian(s). Ethical approval was provided by the Ethical Review Committee of the Ministry of Health, Burkina Faso (No. 2022-05-093), Institutional Ethics Review Committee for Health Science Research Bobo Dioulasso (A014-2022-CEIRES). For the cohort study in Uganda, ethical approval was obtained from the Makerere University School of Medicine Research and Ethics Committee, the Uganda National Council for Science and Technology, the London School of Hygiene & Tropical Medicine Ethics Committee, the Durham University School of Biological and Biomedical Sciences Ethics Committee, and the University of California, San Francisco, Committee on Human Research.

### Statistical analysis

TRA was calculated as the reduction in oocysts compared to a negative control using an online tool (*38*). All other statistical analyses were performed using GraphPad Prism (version 10.1.0).

## Supplementary Materials

### The PDF file includes:

Figs. S1 to S6 Tables S1 to S4 Legend for data S1 References
