# Supplementary material for: Pfs230 domain 12 is a potent malaria transmission–blocking vaccine candidate
Source: Sci Adv. 2025 Nov 7;11(45):eadw8216. doi: 10.1126/sciadv.adw8216 (PMC12594191; doi:10.1126/sciadv.adw8216)
Supplement: Supplementary file 1 — Figs. S1 to S6 Tables S1 to S4 Legend for data S1 References [file sciadv.adw8216_sm.pdf]

Science Advances
AAAS

Supplementary Materials for

Pfs230 domain 12 is a potent malaria transmission–blocking vaccine candidate

Maartje R. Inklaar *et al.*

Corresponding author: Matthijs M. Jore, matthijs.jore@radboudumc.nl

Sci. Adv. **11**, eadw8216 (2025)
DOI: 10.1126/sciadv.adw8216

The PDF file includes:

Figs. S1 to S6
Tables S1 to S4
Legend for data S1
References

Other Supplementary Material for this manuscript includes the following:

Data S1

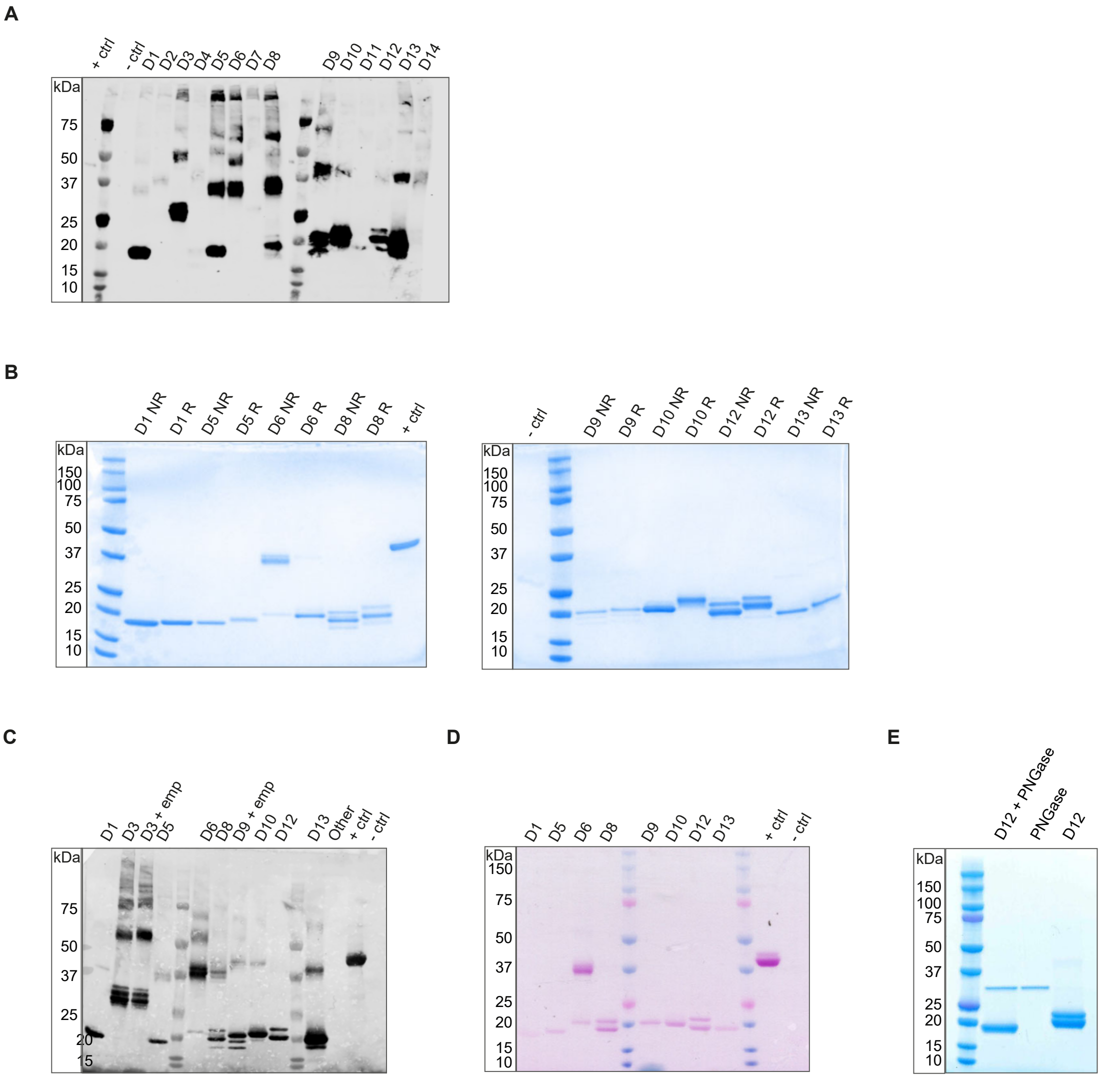

Fig. S1.

Analysis of recombinant Pfs230 domain protein constructs produced in S2 cells. (A) Western blot analysis of S2 cell supernatants. Supernatants of D1 till D14 were harvested from stable transfected cell lines. Supernatants were separated by SDS-PAGE, transferred to western blot, and stained with 1:1000 C-tag antibody and 1:2500 IRDye Streptavidin 680LT. + ctrl: Pro-CS3-6C; - ctrl: supernatant from un-transfected cells. **(B)** Coomassie-stained SDS-PAGE gels of Pfs230 single domain constructs purified from S2 cells and used for mice immunizations (original version of Figure 1B). NR: non-reduced conditions; R: reduced conditions. **(C)** Western blot of single domain Pfs230 constructs used for mice immunizations (original version of Figure 1C). **(D)** Glycoprotein staining after SDS-page (original version of Figure 1D). (**E**) Coomassie-stained SDS-PAGE gel of deglycosylated D12. D12 was deglycosylated with PNGase F under denaturing and reducing conditions and all samples were analyzed on gel under reducing conditions.

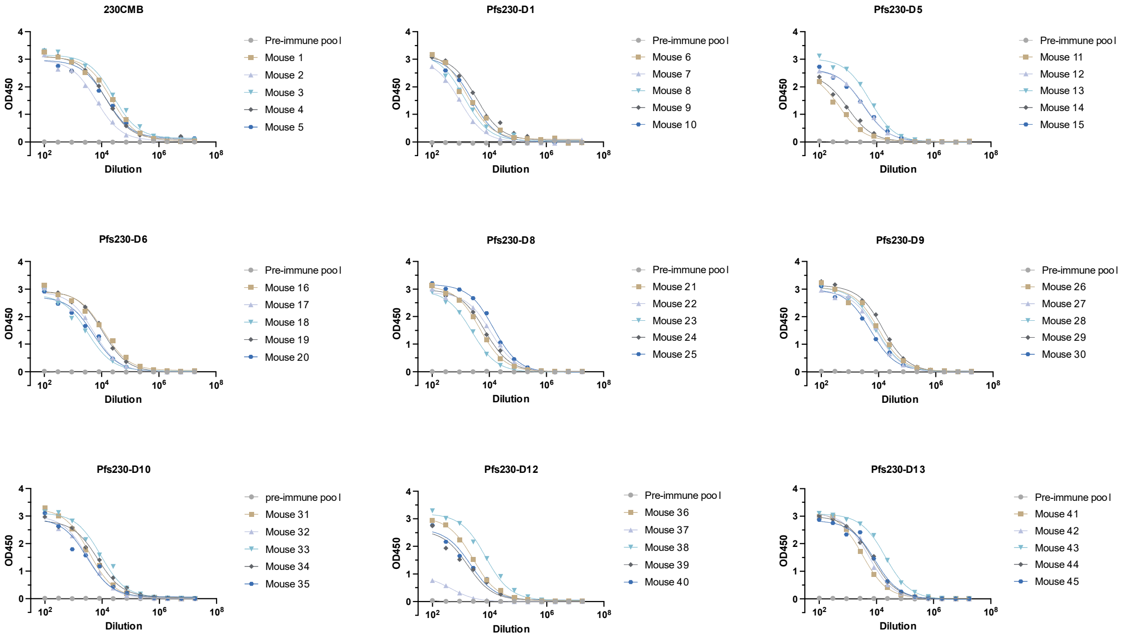

Fig. S2.

Antigen specific ELISAs with sera from immunized mice. The sera of each individual mouse were titrated (12-point titration, in singlicate) along with the pooled pre-immune sera. Sigmoidal curve fits were used to calculate EC_{50} values that are shown in figure 2B.

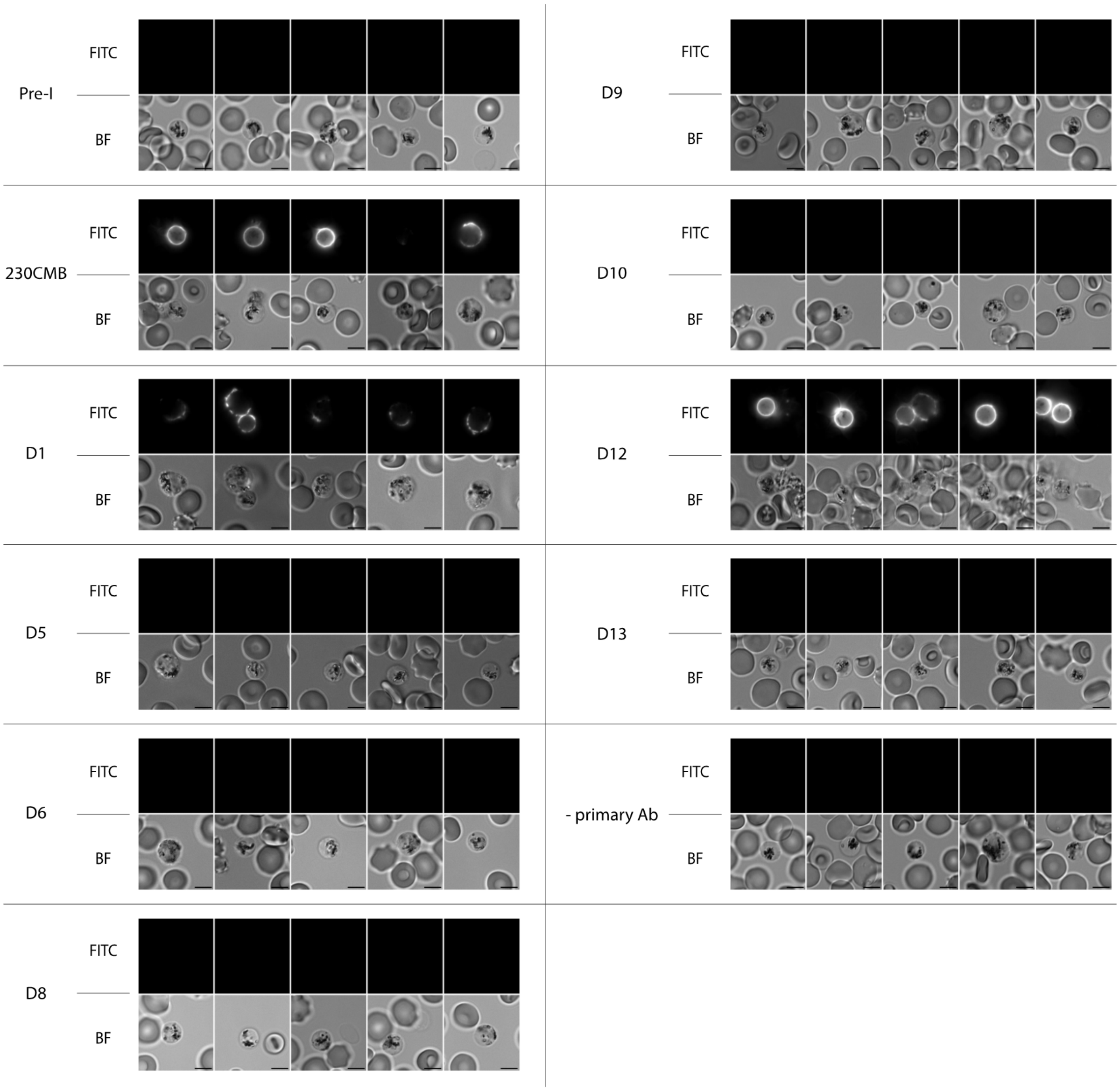

Fig. S3.

Recognition of female gamete surface by mouse antibodies. Pooled mouse serum was tested at 1:100 dilution in surface immuno fluorescence assay (SIFA) with live female gametes. Bound mouse antibodies were detected with a FITC labeled secondary antibody. BF = bright field image. Scale bar indicates 5 µm. Pre-I = pre-immune serum.

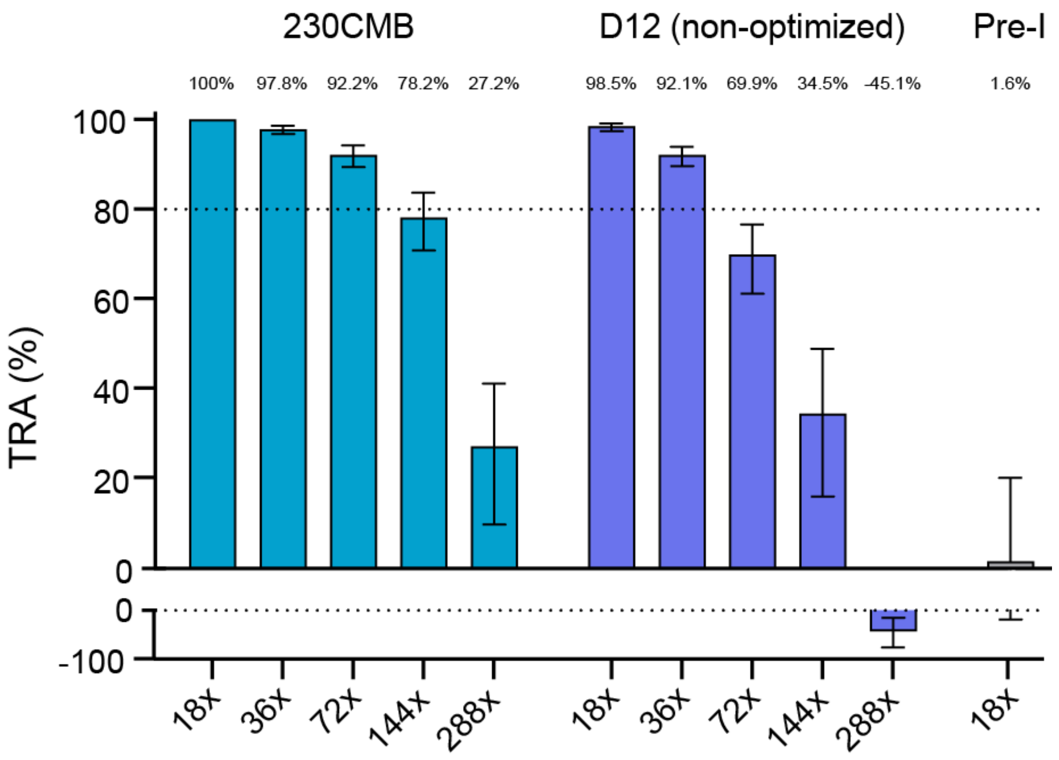

Fig. S4.

SMFA with dilution series of 230CMB and D12 serum. Pooled mouse serum was tested in a dilution series, with each dilution tested in at least two independent SMFA experiments. Dilution factors are shown below the graph. Bars and values above indicate the estimated TRA. Error bars indicate the 95% confidence intervals. Pre-I = pre-immune serum. Raw oocyst count data are provided in Data S1.

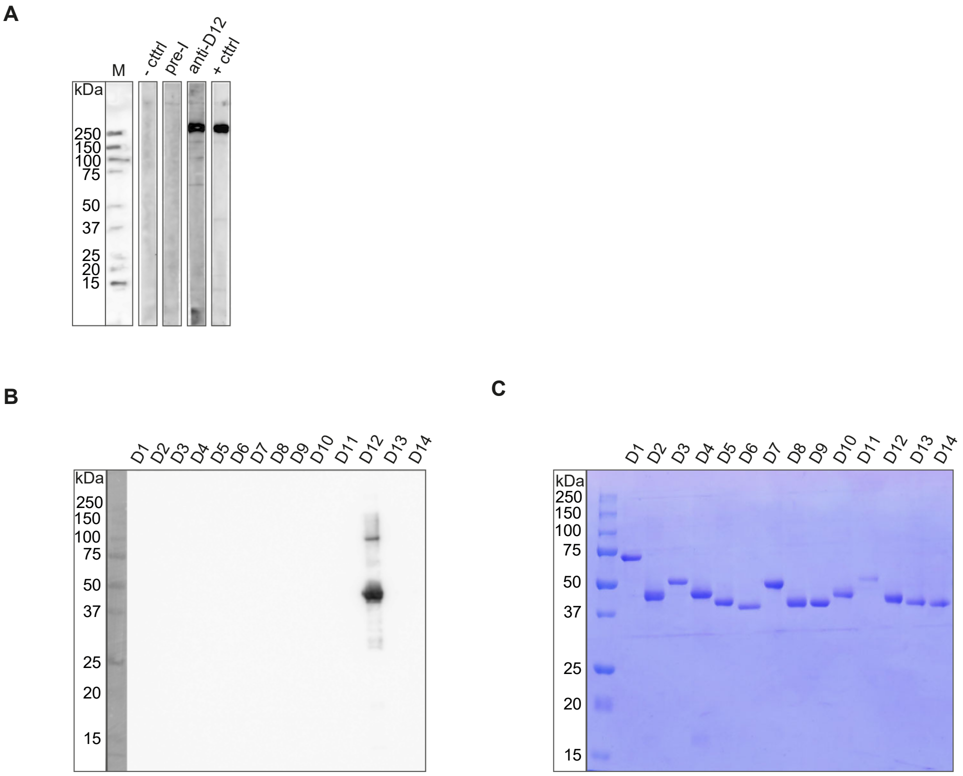

Fig. S5.

Pfs230 and Pfs230-D12 specificity of the Pfs230-D12 induced antibodies. Western blots incubated with mice sera induced by S2 cell-produced Pfs230-D12 showing specific recognition of **(A)** Pfs230 present in gametocyte extract and of **(B)** Pfs230-D12 recombinant protein produced by the wheat germ cell-free system. **(C)** The Pfs230 domains were separated by SDS-PAGE followed by Coomassie brilliant blue staining.

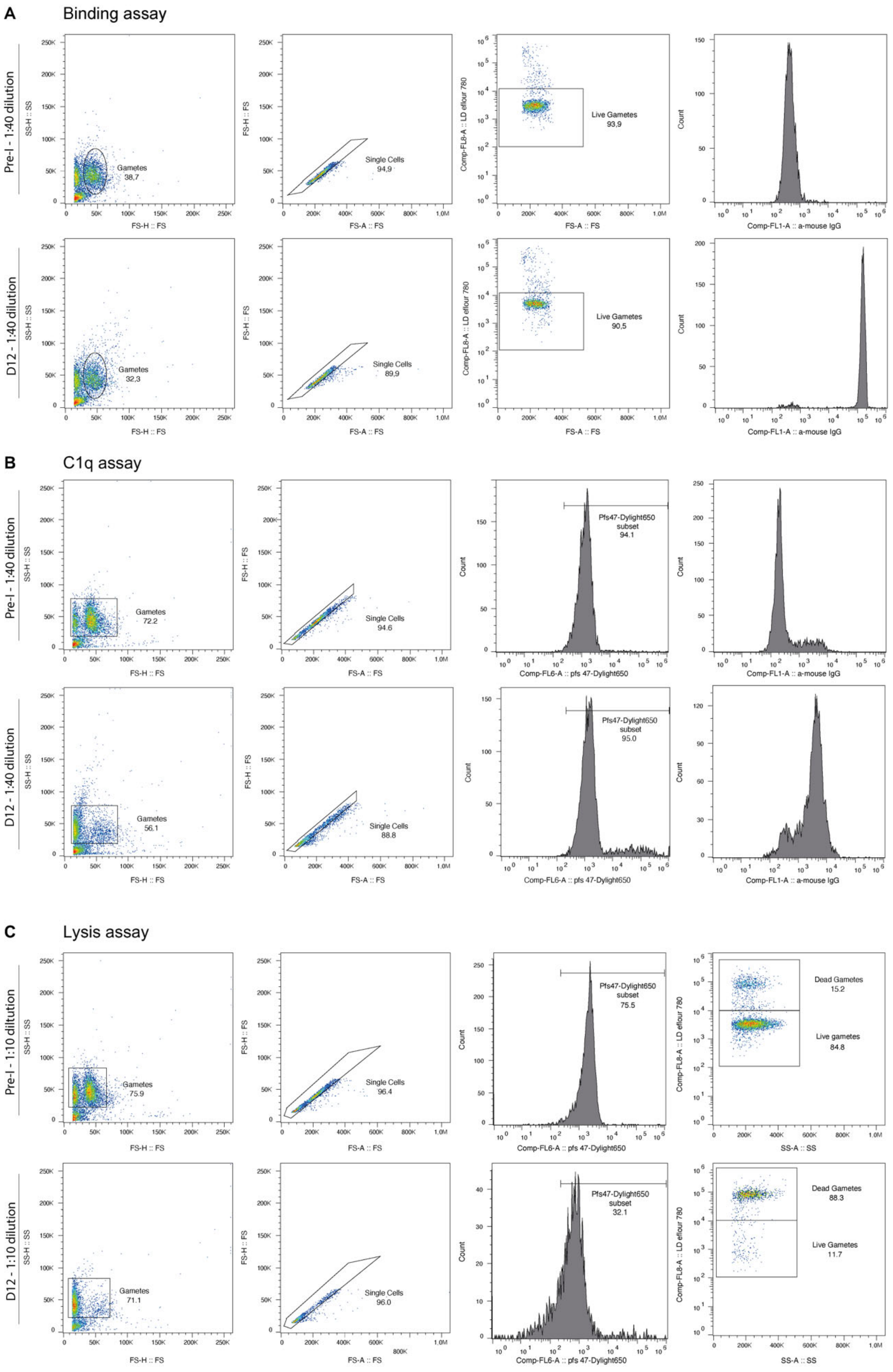
A
Binding assay
Pre-I - 1:40 dilution
D12 - 1:40 dilution
Gametes
38,7
32,3
Single Cells
94,9
89,9
Live Gametes
93,9
90,5
SS-H :: SS
FS-H :: FS
FS-A :: FS
Comp-FL8-A :: LD eflour 780
Count
Comp-FL1-A :: a-mouse IgG
B
C1q assay
Pre-I - 1:40 dilution
D12 - 1:40 dilution
Gametes
72.2
56.1
Single Cells
94.6
88.8
Pfs47-Dylight650 subset
94.1
95.0
Comp-FL6-A :: pfs 47-Dylight650
Comp-FL1-A :: a-mouse IgG
C
Lysis assay
Pre-I - 1:10 dilution
D12 - 1:10 dilution
Gametes
75.9
71.1
Single Cells
96.4
96.0
Pfs47-Dylight650 subset
75.5
32.1
Dead Gametes
15.2
88.3
Live gametes
84.8
Live Gametes
11.7
SS-A :: SS

Fig. S6.

Overview of flow cytometry gating strategies. Exemplary plots that provide an overview of the gating strategy for **(A)** an antibody binding assay, **(B)** a C1q fixation assay and **(C)** a lysis assay, all with live female gametes. For each of the assays a representative set of plots is shown for pre-immune serum and D12 serum. In the binding assay **(A)** gametes were gated for single cells (2nd column) and then live gametes were selected based on the absence of live-dead stain LD efluor 780, which stains dead cells (3rd column). For the C1q deposition assay **(B)** the gated single cells from the 2nd column were gated for gamete marker Pfs47 positivity, to then determine the anti-C1q deposition by FITC-labelled anti-C1q staining. For the lysis assay **(C)** gametes were gated for single cells (2nd column), and then for Pfs47 positivity. Dead gametes were stained with live-dead stain LD efluor 780 to determine the percentage dead cells (4th column). Note that two gamete populations can be observed in the forward scatter plots (1st column) in A and B; the left population contains dead gametes, the right population contains live gametes. In the antibody binding assay (A) and C1q deposition assay (B) we gated only the live population, while in the lysis assay (C) we gated both live and dead populations. Pre-I = pre-immune serum. SS-H = side scatter height, SS = side scatter, FS-H = forward scatter height, FS-A = forward scatter area, LD = live dead.

Table S1.

Overview of Pfs230 single domain sequences as they are expressed in S2 cells.

**Pfs230 single domain**	**Nucleotide sequence**<br>Underlined = kozak sequence<br>*Italic* = signal peptide<br>**Blue = His-tag**<br>**Dark grey = linker (also restriction site Ngo MIV)**<br>**Grey = domain**<br>**Yellow = linker**<br>**Dark blue = C-tag**
D1	GCCACC*ATGAAGCTGTGCATCCTGCTGGCCGTGGTGGCCTTCGTGGGACTGAGCCTGGGA*CACC<br>ACCACCATCACCACGCCGGCAAAGAGTACGTCTGCGACTTCACCGATCAGCTGAAGCCAACCGA<br>GTCGGGCCCCAAAGTGAAGAAATGCGAAGTGAAAGTGAACGAGCCCCTGATCAAAGTCAAGATT<br>ATCTGCCCGCTGAAGGGCAGCGTGGAAAAGCTGTACGATAACATCGAGTACGTGCCCAAGAAAA<br>GCCCCTACGTGGTGCTGACCAAAGAGGAAACGAAGCTGAAAGAGAAGCTGCTGAGCAAGCTGAT<br>CTACGGCCTGCTGATCTCCCCGACCGTGAACGAGAAAGAGAACAACTTCAAAGAGGGCGTCATC<br>GAGTTCACCCTGCCGCCAGTGGTGCATAAGGCCACCGTGTTCTACTTCATCTGCGACAACAGCA<br>AGACCGAGGACGATAACAAGAAGGGCAACCGCGGCATCGTGGAAGTGTACGTGGAACCCTACGG<br>ATCAGAGCCCGAGGCCTAA
D2	GCCACC*ATGAAGCTGTGCATCCTGCTGGCCGTGGTGGCCTTCGTGGGACTGAGCCTGGGA*CACC<br>ACCACCATCACCACGCCGGCGGCAACAAGATCAACGGCTGCGCCTTCCTGGATGAGGATGAGGA<br>AGAAGAGAAGTACGGCAATCAGATCGAAGAGGACGAGCACAACGAGAAGATCAAGATGAAGACC<br>TTCTTCACCCAAAACATCTACAAGAAGAACAACATCTACCCGTGCTACATGAAGCTGTACTCCG<br>GCGATATCGGCGGCATTCTGTTCCCCAAGAACATCAAGAGCACGACCTGCTTCGAGGAAATGAT<br>CCCCTACAACAAAGAAATCAAGTGGAACAAAGAGAACAAGAGCCTGGGCAACCTGGTCAACAAC<br>AGCGTGGTGTATAACAAAGAGATGAACGCCAAGTACTTCAACGTGCAGTACGTGCACATCCCCA<br>CCAGCTACAAGGATACCCTGAACCTGTTCTGCAGCATCATCCTGAAAGAGGAAGAGAGCAACCT<br>GATCAGCACCTCCTACCTGGTGTACGTGTCCATCAACGAGGGATCAGAGCCCGAGGCCTAA
D3	GCCACC*ATGAAGCTGTGCATCCTGCTGGCCGTGGTGGCCTTCGTGGGACTGAGCCTGGGA*CACC<br>ACCACCATCACCACGCCGGCCACGATTATACCTGCGATTTCACGGACAAGCTCGACAAGACCGT<br>GCCGAGCACCGCCAATGGCAAGAAGCTGTTCATCTGCCGCAAGCACCTGAAAGAATTCGACACC<br>TTCACGCTGAAGTGCAACGTGAACAAGACGCAGTACCCCAACATCGAGATCTTCCCAAAGACGC<br>TGAAGGACAAGAAAGAGGTCCTGAAGCTGGATCTGGACATCCAGTACCAGATGTTCAGCAAGTT<br>CTTCAAGTTTAACACCCAGAACGCGAAGTACCTGAATCTGTACCCCTACTACCTGATCTTCCCC<br>TTCAACCACATCGGAAAGAAAGAGCTGAAAAACAACCCCACCTACAAGAACCACAAGGACGTGA<br>AGTATTTCGAGCAGTCCTCCGTGCTGAGCCCACTGAGTAGTGCCGATAGCCTGGGAAAGCTGTT<br>GAACTTCCTGGACACCCAAGAGACAGTGTGCCTGACCGAGAAGATTCGCTATCTGAACCTGAGC<br>ATCAATGAGCTGGGCAGCGATAACAACACCTTCTCCGTGACGTTCCAGGTGCCGCCGTACATCG<br>ATATCAAAGAACCCTTCTACTTTATGTTCGGCTGCAACAACAACAAAGGCGAGGGCAACATCGG<br>CATAGTCGAGCTGCTGATTAGCAAGCAAGGATCAGAGCCCGAGGCCTAA
D4	GCCACC*ATGAAGCTGTGCATCCTGCTGGCCGTGGTGGCCTTCGTGGGACTGAGCCTGGGA*CACC<br>ACCACCATCACCACGCCGGCGAAGAAAAGATTAAGGGCTGCAATTTCCACGAGTCCAAGCTGGA<br>CTACTTCAATGAGAACATCAGCAGCGATACCCACGAGTGCACGCTGCACGCCTATGAGAACGAT<br>ATCATCGGCTTCAACTGCCTGGAAACGACGCACCCCAACGAGGTGGAAGTGGAAGTTGAGGATG<br>CCGAGATCTATCTGCAGCCCGAGAACTGCTTCAACAACGTCTACAAGGGCCTGAACTCCGTGGA<br>TATCACCACCATCCTGAAGAACGCCCAGACCTACAACATTAACAACAAAAAGACCCCGACCTTC<br>CTGAAGATCCCGCCATACAACCTGCTGGAAGATGTGGAAATCAGCTGCCAGTGCACCATCAAGC<br>AAGTGGTCAAGAAAATCAAAGTGATCATCACCAAGAACGACGGATCAGAGCCCGAGGCCTAA
D5	GCCACC*ATGAAGCTGTGCATCCTGCTGGCCGTGGTGGCCTTCGTGGGACTGAGCCTGGGA*CACC<br>ACCACCATCACCACGCCGGCAAGATCTACAAGTGCGAGCACGAGAACTTCATCAACCCGCGCGT

	CAACAAGACCTTCGACGAGAACGTCGAGTACACGTGCAATATCAAGATCGAGAATTTCTTCAAC<br>TACATCCAGATTTTCTGCCCCGCCAAGGATCTGGGCATCTATAAGAATATCCAGATGTACTACG<br>ACATCGTGAAGCCGACGCGCGTGCCCCAGTTCAAAAAATTCAACAATGAGGAGCTCCACAAGCT<br>CATCCCCAACTCCGAGATGCTGCACAAGACGAAAGAGATGCTGATCCTGTACAACGAAGAGAAG<br>GTGGACCTGCTGCACTTCTACGTGTTCTTGCCCATCTACATCAAGGACATCTACGAGTTCAACA<br>TCGTGTGCGACAACTCCAAGACGATGTGGAAGAACCAGCTCGGCGGAAAAGTGATCTACCACAT<br>CACCGTCAGCAAGCGCGGATCAGAGCCCGAGGCCTAA
D6	GCCACC*ATGAAGCTGTGCATCCTGCTGGCCGTGGTGGCCTTCGTGGGACTGAGCCTGGGA*CACC<br>ACCACCATCACCACGCCGGCTTCGATAACGAGCACGCCCACATGTTCAGCTATAACAAGACCAA<br>CGTGAAGAACTGCATCATCGACGCCAAGCCGAAGGATCTGATCGGCTTCGTGTGCCCAAGCGGC<br>ACACTGAAGCTGACCAATTGCTTCAAGGATGCCATCGTGCACACCAACCTGACCAACATCAACG<br>GCATCCTGTATCTCAAGAACAACCTGGCCAACTTCACGTACAAGCACCAGTTCAATTACATGGA<br>AATCCCCGCGCTGATGGACAACGACATCAGCTTCAAGTGCATCTGCGTGGACCTGAAGAAAAAG<br>AAGTACAACGTCAAGAGCCCGCTGGGCCCCGGATCAGAGCCCGAGGCCTAA
D7	GCCACC*ATGAAGCTGTGCATCCTGCTGGCCGTGGTGGCCTTCGTGGGACTGAGCCTGGGA*CACC<br>ACCACCATCACCACGCCGGCAACCGCCACGTGTGCGATTTCTCCAAGAACAATCTGATCGTGCC<br>CGAGTCGTTGAAGAAGAAAGAGGAACTCGGCGGCAACCCCGTGAACATCCATTGCTATGCCCTG<br>TTGAAGCCCCTGGATACGCTGTATGTGAAGTGCCCCACCTCCAAGGATAACTACGAGGCCGCCA<br>AAGTCAACATCAGCGAGAATGATAACGAGTACGAGTTGCAAGTGATCTCCCTGATCGAGAAGCG<br>CTTTCACAACTTCGAGACACTGGAAAGCAAAAAGCCCGGCAACGGCGACGTCGTGGTGCACAAT<br>GGTGTTGTGGATACCGGACCGGTGCTGGATAACTCCACGTTCGAGAAGTACTTTAAGAACATTA<br>AGATCAAGCCCGATAAGTTCTTCGAGAAAGTTATCAATGAGTACGACGACACCGAGGAAGAAAA<br>GGACCTGGAATCCATCCTGCCAGGCGCCATCGTGTCCCCAATGAAGGTGCTCAAGAAGAAGGAC<br>CCCTTCACCAGCTATGCCGCCTTTGTGGTGCCACCGATCGTGCCAAAGGATCTGCACTTCAAGG<br>TGGAATGCAACAATACCGAGTACAAGGACGAGAACCAGTACATCAGCGGCTACAATGGCATCAT<br>CCACATCGACATCTCCAACAGCGGATCAGAGCCCGAGGCCTAA
D8	GCCACC*ATGAAGCTGTGCATCCTGCTGGCCGTGGTGGCCTTCGTGGGACTGAGCCTGGGA*CACC<br>ACCACCATCACCACGCCGGCAACCGCAAGATCAATGGATGCGACTTTAGCACCAACAACTCCAG<br>CATCCTGACCAGCTCCGTGAAGCTGGTTAACGGCGAGACAAAGAACTGCGAGATCAATATCAAC<br>AACAACGAAGTGTTCGGCATCATCTGTGACAATGAGACAAATCTGGACCCAGAGAAGTGCTTCC<br>ATGAGATCTACTCCAAGGACAACAAGACGGTCAAAAAGTTCCGCGAAGTGATCCCCAATATCGA<br>CATTTTCAGCCTGCACAACTCGAACAAGAAAAAGGTGGCCTACGCCAAGGTGCCCCTGGACTAT<br>ATTAACAAGCTGCTGTTCAGCTGCTCCTGCAAGACCAGCCACACCAACACCATCGGCACGATGA<br>AAGTGACCCTGAACAAAGACGAGGGATCAGAGCCCGAGGCCTAA
D9	GCCACC*ATGAAGCTGTGCATCCTGCTGGCCGTGGTGGCCTTCGTGGGACTGAGCCTGGGA*CACC<br>ACCACCATCACCACGCCGGCAACGTGCACCTGTGCAATTTCTTCGACAACCCCGAGCTGACCTT<br>CGACAACAACAAGATCGTGCTGTGCAAGATCGATGCCGAGCTGTTTAGCGAAGTCATCATTCAG<br>CTGCCCATCTTCGGCACCAAAAACGTCGAGGAAGGCGTCCAGAACGAAGAGTACAAGAAGTTCA<br>GCCTGAAGCCGAGCCTGGTGTTCGATGATAACAACAATGACATCAAAGTCATCGGCAAAGAGAA<br>GAACGAGGTTTCCATCTCGCTGGCCCTGAAGGGCGTGTACGGCAACCGCATCTTCACCTTTGAT<br>AAGAACGGCAAGAAAGGCGAAGGCATCAGCTTTTTCATCCCGCCGATCAAGCAGGATACCGACC<br>TGAAGTTTATCATCAACGAAACCATCGATAACAGCAACATTAAGCAGCGCGGCCTGATCTACAT<br>CTTCGTGCGCAAGAACGTGTCGGGATCAGAGCCCGAGGCCTAA
D10	GCCACC*ATGAAGCTGTGCATCCTGCTGGCCGTGGTGGCCTTCGTGGGACTGAGCCTGGGA*CACC<br>ACCACCATCACCACGCCGGCGAGAACTCGTTCAAGCTGTGTGATTTCACCACCGGCAGCACCAG<br>CCTGATGGAATTGAACAGCCAAGTGAAAGAAAAGAAGTGCACCGTTAAGATTAAGAAGGGCGAT<br>ATCTTCGGCCTGAAATGCCCCAAGGGATTCGCCATTTTTCCGCAAGCCTGCTTCTCCAACGTCC<br>TGCTCGAGTACTACAAGAGCGATTACGAGGACAGCGAGCACATCAACTACTACATTCACAAGGA<br>CAAAAAGTACAATCTGAAGCCCAAGGACGTTATCGAGTTGATGGATGAGAACTTCCGCGAGCTG<br>CAAAACATTCAGCAGTACACCGGCATCAGCAACATCACCGATGTGCTGCATTTCAAGAACTTCA<br>ACCTGGGCAATCTGCCGCTCAACTTCAAGAATCACTACAGCACCGCCTATGCGAAGGTGCCGGA

	TACCTTCAACTCCATCATCAACTTCAGCTGCAACTGCTACAATCCCGAGAAGCACGTCTACGGC<br>ACCATGCAGGTCGAGAGCGATAACGGATCAGAGCCCGAGGCCTAA
D11	GCCACC*ATGAAGCTGTGCATCCTGCTGGCCGTGGTGGCCTTCGTGGGACTGAGCCTGGGA*CACC<br>ACCACCATCACCACGCCGGCAATGAGCACATTTGCGACTACGAAAAGAACGAGTCGCTGATCTC<br>GACCCTGCCAAACGACACCAAGAAGATCCAGAAGTCGATCTGCAAGATTAACGCGAAGGCCCTG<br>GATGTGGTCACCATTAAGTGCCCGCATACCAAGAATTTCACCCCGAAGGATTACTTCCCCAACA<br>GCAGCCTGATCACCAACGATAAGAAGATCGTCATCACGTTCGATAAGAAAAACTTCGTCACCTA<br>CATCGACCCCACCAAAAAGACGTTCTCCCTGAAAGACATCTACATTCAGAGCTTCTACGGCGTG<br>TCCCTGGATCACCTGAACCAGATCAAAAAAATCCACGAGGAATGGGACGACGTCCACCTGTTTT<br>ACCCGCCGCACAACGTTCTGCACAACGTGGTCCTGAACAACCACATTGTGAACCTGTCCAGCGC<br>CTTGGAGGGCGTGCTGTTCATGAAGTCCAAAGTGACCGGCGACGAGACAGCCACGAAGAAGAAT<br>ACCACACTGCCCACCGATGGCGTGTCCAGCATTCTGATCCCGCCGTACGTGAAAGAAGATATCA<br>CCTTCCATCTGTTCTGCGGCAAGTCCACGACCAAGAAGCCCAACAAAAAAAACACCAGCTTGGC<br>CCTGATCCACATTCACATCAGCTCCAATGGATCAGAGCCCGAGGCCTAA
D12	GCCACC*ATGAAGCTGTGCATCCTGCTGGCCGTGGTGGCCTTCGTGGGACTGAGCCTGGGA*CACC<br>ACCACCATCACCACGCCGGCCGCAATATCATCCACGGCTGCGACTTTCTGTACCTGGAAAACCA<br>GACCAACGACGCCATCTCGAACAACAACAACAACTCCTACAGCATCTTCACCCACAACAAGAAC<br>ACCGAGAACAACCTCATCTGCGATATTTCGCTGATCCCCAAGACCGTGATCGGCATCAAGTGCC<br>CCAACAAGAAGCTGAACCCGCAGACCTGCTTTGACGAGGTGTACTACGTCAAACAAGAGGACGT<br>GCCGTCCAAGACCATCACCGCCGACAAGTACAATACCTTCAGCAAGGATAAGATTGGCAACATC<br>CTCAAAAACGCCATCAGCATCAACAACCCGGACGAGAAGGATAACACCTACACCTATCTGATCC<br>TGCCGGAAAAGTTCGAGGAAGAGTTGATCGATACCAAAAAGGTGCTGGCCTGCACGTGTGACAA<br>CAAGTACATTATCCACATGAAGATCGAAAAGTCCACCGGATCAGAGCCCGAGGCCTAA
D13	GCCACC*ATGAAGCTGTGCATCCTGCTGGCCGTGGTGGCCTTCGTGGGACTGAGCCTGGGA*CACC<br>ACCACCATCACCACGCCGGCGGCAAGGATATCTGCAAATACGACGTGACCACCAAGGTGGCCAC<br>GTGCGAGATTATCGACACCATCGATTCGAGCGTGCTGAAAGAACACCACACCGTGCACTACTCG<br>ATCACCCTGTCGCGCTGGGATAAGCTGATCATCAAGTACCCGACCAACGAGAAAACCCACTTTG<br>AGAACTTTTTCGTGAACCCGTTCAACCTCAAGGACAAGGTGCTCTACAATTACAACAAGCCCAT<br>CAACATTGAGCACATACTGCCCGGTGCCATCACCACCGATATCTACGATACGCGCACCAAGATT<br>AAGCAGTACATCCTGCGCATCCCACCGTATGTGCACAAGGATATTCACTTCTCCCTGGAATTCA<br>ACAACTCCCTGAGCCTGACCAAGCAGAACCAGAACATTATCTACGGCAATGTGGCCAAGATCTT<br>CATCCATATCAACCAGGGCGGATCAGAGCCCGAGGCCTAA
D14	GCCACC*ATGAAGCTGTGCATCCTGCTGGCCGTGGTGGCCTTCGTGGGACTGAGCCTGGGA*CACC<br>ACCACCATCACCACGCCGGCTACAAAGAGATCCACGGTTGCGATTTCACCGGCAAGTACAGCCA<br>CCTGTTCACCTACTCCAAAAAGCCGCTGCCGAACGATGACGACATCTGCAATGTGACCATCGGA<br>AACAACACGTTCAGCGGATTCGCCTGCCTGTCGCACTTCGAGCTGAAACCCAACAACTGCTTCT<br>CGTCGGTGTACGATTACAACGAGGCCAACAAAGTGAAAAAGTTGTTCGACCTGTCGACCAAGGT<br>CGAGCTGGATCACATCAAACAGAACACCTCCGGCTACACCCTGTCCTACATCATTTTTAACAAA<br>GAATCGACCAAGCTCAAGTTCTCCTGCACATGCAGCAGCAACTACTCCAACTACACCATCCGCA<br>TCACCTTCGATCCGGGATCAGAGCCCGAGGCCTAA

Table S2.

Characteristics of all Pfs230 single domain constructs. NetOGlyc version 4.0 (DTU Health Tech, Denmark) (*38*) and NetNglyc version 1.0 (DTU Health Tech, Denmark) (*39*) were used to predict the O-linked and N-linked glycosylation sites.

Pfs230 single Domain	Amino acid numbers within full-length Pfs230	Molecular weight (kDa)	Number of cysteines	Predicted N-linked glycosylation sites	Predicted O-linked glycosylation sites
D1	589-730	17.8	4	0	2
D2	731-886	19.8	4	2	0
D3	918-1133	26.8	5	4	2
D4	1134-1268	17.1	6	1	0
D5	1285-1432	19.5	4	1	0
D6	1433-1550	14.1	6	2	0
D7	1694-1907	25.9	4	3	1
D8	1908-2036	16.1	6	4	1
D9	2052-2201	18.7	2	1	0
D10	2202-2373	21.5	6	2	0
D11	2448-2663	26.1	4	7	4
D12	2664-2818	19.4	6	1	0
D13	2831-2979	19.0	2	0	0
D14	2980-3105	16.0	6	5	0

Table S3.

Mass spectrometry results confirm Pfs230-D12 identity. The table includes all Pfs230 recombinant domains produced in this study that were found in the peptide preparation.

Protein ID	Intensity	% of total intensity	Peptides	Razor + unique peptides	Unique peptides	% coverage
Pfs230-D12	9.853×10^{11}	98.226	29	29	29	89.9
Pfs230-D5	5.715×10^{6}	0.001	2	2	2	15.6

Table S4.

Peptides, covering recombinant Pfs230 fragments, identified by mass spectrometry.

Domain	Peptide
D12	CPNKKLNPQTCFDEVYYVK
D12	DKIGNILK
D12	DNTYTYLILPEK
D12	DNTYTYLILPEKFEEELIDTK
D12	DNTYTYLILPEKFEEELIDTKK
D12	FEEELIDTK
D12	FEEELIDTKK
D12	KLNPQTCFDEVYYVK
D12	KLNPQTCFDEVYYVKQEDVPSK
D12	KVLACTCDNK
D12	KVLACTCDNKYIIHMK
D12	LNPQTCFDEVYYVK
D12	LNPQTCFDEVYYVKQEDVPSK
D12	NAISINNPDEK
D12	NAISINNPDEKDNTYTYLILPEK
D12	NAISINNPDEKDNTYTYLILPEKFEEELIDTK
D12	NIIHGCDFLYLENQTNDAISNNNNNSYSIFTHNK
D12	NTENNLICDISLIPK
D12	NTENNLICDISLIPKTVIGIK
D12	QEDVPSK
D12	QEDVPSKTITADKYNTFSK
D12	TITADKYNTFSK
D12	TITADKYNTFSKDK
D12	VLACTCDNK
D12	VLACTCDNKYIIHMK
D12	VLACTCDNKYIIHMKIEK
D12	YIIHMKIEK
D12	YNTFSKDK
D5	IENFFNYIQIFCPAK
D5	TFDENVEYTCNIK

Data S1.

Raw SMFA and DMFA data

REFERENCES AND NOTES

1. WHO, *World Malaria Report 2023* (World Health Organization, 2023).

2. P. L. Alonso, G. Brown, M. Arevalo-Herrera, F. Binka, C. Chitnis, F. Collins, O. K. Doumbo, B. Greenwood, B. F. Hall, M. M. Levine, K. Mendis, R. D. Newman, C. V. Plowe, M. H. Rodríguez, R. Sinden, L. Slutsker, M. Tanner, A research agenda to underpin malaria eradication. *PLOS Med.* **8**, e1000406 (2011).

3. S. Eksi, B. Czesny, G. J. van Gemert, R. W. Sauerwein, W. Eling, K. C. Williamson, Malaria transmission-blocking antigen, Pfs230, mediates human red blood cell binding to exflagellating male parasites and oocyst production. *Mol. Microbiol.* **61**, 991–998 (2006).

4. F. M. T. Lyons, M. Gabriela, W. H. Tham, M. H. Dietrich, *Plasmodium* 6-cysteine proteins: Functional diversity, transmission-blocking antibodies and structural scaffolds. *Front. Cell. Infect. Microbiol.* **12**, 945924 (2022).

5. D. L. Gerloff, A. Creasey, S. Maslau, R. Carter, Structural models for the protein family characterized by gamete surface protein Pfs230 of *Plasmodium falciparum*. *Proc. Natl. Acad. Sci. U.S.A.* **102**, 13598–13603 (2005).

6. P. E. Duffy, The virtues and vices of Pfs230: From vaccine concept to vaccine candidate. *Am. J. Trop. Med. Hyg.* **107**, 17–21 (2022).

7. P. J. Bustamante, D. C. Woodruff, J. Oh, D. B. Keister, O. Muratova, K. C. Williamson, Differential ability of specific regions of *Plasmodium falciparum* sexual-stage antigen, Pfs230, to induce malaria transmission-blocking immunity. *Parasite Immunol.* **22**, 373–380 (2000).

8. K. C. Williamson, D. B. Keister, O. Muratova, D. C. Kaslow, Recombinant Pfs230, a *Plasmodium falciparum* gametocyte protein, induces antisera that reduce the infectivity of *Plasmodium falciparum* to mosquitoes. *Mol. Biochem. Parasitol.* **75**, 33–42 (1995).

9. M. Tachibana, K. Miura, E. Takashima, M. Morita, H. Nagaoka, L. Zhou, C. A. Long, C. Richter King, M. Torii, T. Tsuboi, T. Ishino, Identification of domains within Pfs230 that elicit transmission blocking antibody responses. *Vaccine* **37**, 1799–1806 (2019).

10. K. Miura, E. Takashima, T. P. Pham, B. Deng, L. Zhou, W. C. Huang, A. Diouf, Y. T. Gebremicale, M. Tachibana, T. Ishino, C. Richter King, J. F. Lovell, C. A. Long, T. Tsuboi, Elucidating functional epitopes within the N-terminal region of malaria transmission blocking vaccine antigen Pfs230. *NPJ Vaccines* **7**, 4 (2022).

11. M. Tachibana, Y. Wu, H. Iriko, O. Muratova, N. J. MacDonald, J. Sattabongkot, S. Takeo, H. Otsuki, M. Torii, T. Tsuboi, N-terminal prodomain of Pfs230 synthesized using a cell-free system is sufficient to induce complement-dependent malaria transmission-blocking activity. *Clin. Vaccine Immunol.* **18**, 1343–1350 (2011).

12. N. J. MacDonald, V. Nguyen, R. Shimp, K. Reiter, R. Herrera, M. Burkhardt, O. Muratova, K. Kumar, J. Aebig, K. Rausch, L. Lambert, N. Dawson, J. Sattabongkot, X. Ambroggio, P. E. Duffy, Y. Wu, D. L. Narum, Structural and immunological characterization of recombinant 6-cysteine domains of the *Plasmodium falciparum* sexual stage protein Pfs230. *J. Biol. Chem.* **291**, 19913–19922 (2016).

13. S. A. Healy, C. Anderson, B. J. Swihart, A. Mwakingwe, E. E. Gabriel, H. Decederfelt, C. V. Hobbs, K. M. Rausch, D. Zhu, O. Muratova, R. Herrera, P. V. Scaria, N. J. MacDonald, L. E. Lambert, I. Zaidi, C. H. Coelho, J. P. Renn, Y. Wu, D. L. Narum, P. E. Duffy, Pfs230 yields higher malaria transmission-blocking vaccine activity than Pfs25 in humans but not mice. *J. Clin. Invest.* **131**, (2021).

14. I. Sagara, S. A. Healy, M. H. Assadou, M. Kone, B. J. Swihart, J. L. Kwan, J. Fintzi, K. Sissoko, B. Kamate, Y. Samake, M. A. Guindo, M. Doucoure, K. Niaré, A. Dolo, B. Diarra, K. M. Rausch, D. L. Narum, D. S. Jones, N. J. MacDonald, D. Zhu, J. P. Gorres, A. Imeru, R. Mohan, I. Thera, I. Zaidi, F. Salazar-Miralles, J. Duan, J. Neal, R. D. Morrison, O. Muratova, D. Sylla, E. M. O'Connell, Y. Wu, J. C. C. Hume, M. B. Coulibaly, C. F. Anderson, S. F. Traore, O. K. Doumbo, P. E. Duffy, Malaria transmission-blocking vaccines Pfs230D1-EPA and Pfs25-EPA in Alhydrogel in healthy Malian adults; a phase 1, randomised, controlled trial. *Lancet Infect. Dis.* **23**, 1266–1279 (2023).

15. S. A. Healy, I. Sagara, M. H. Assadou, A. Katile, M. Kone, A. Imeru, J. L. Kwan, B. J. Swihart, J. Fintzi, G. E. Potter, A. Zeguimé, A. Dolo, B. Diarra, D. L. Narum, K. M. Rausch, N. J. M. Donald, D. Zhu, R. Mohan, I. Thera, R. D. Morrison, I. Zaidi, J. Y. A. Doritchamou,

D. Sylla, J. C. C. Hume, M. B. Coulibaly, D. Morelle, M. Lievens, O. K. Doumbo, P. E. Duffy, Pfs230D1 Vaccine Team, A vaccine to block *Plasmodium falciparum* transmission. *NEJM Evid.* **4**, EVIDoa2400188 (2025).

16. L. M. Simons, P. Ferrer, N. Gombakomba, K. Underwood, R. Herrera, D. L. Narum, G. Canepa, F. Acquah, L. Amoah, P. E. Duffy, C. Barillas-Mury, C. Long, S. M. Lee, E. Locke, K. Miura, K. C. Williamson, Extending the range of *Plasmodium falciparum* transmission blocking antibodies. *Vaccine* **41**, 3367–3379 (2023).

17. R. M. de Jong, L. Meerstein-Kessel, D. F. da, S. Nsango, J. D. Challenger, M. van de Vegte-Bolmer, G. J. van Gemert, E. Duarte, N. Teyssier, R. W. Sauerwein, T. S. Churcher, R. K. Dabire, I. Morlais, E. Locke, M. A. Huynen, T. Bousema, M. M. Jore, Monoclonal antibodies block transmission of genetically diverse *Plasmodium falciparum* strains to mosquitoes. *NPJ Vaccines* **6**, 101 (2021).

18. M. R. Inklaar, R. M. de Jong, E. T. Bekkering, H. Nagaoka, F. L. Fennemann, K. Teelen, M. van de Vegte-Bolmer, G. J. van Gemert, R. Stoter, C. R. King, N. I. Proellochs, T. Bousema, E. Takashima, T. Tsuboi, M. M. Jore, Pfs230 Domain 7 is targeted by a potent malaria transmission-blocking monoclonal antibody. *NPJ Vaccines* **8**, 186 (2023).

19. A. Amen, R. Yoo, A. Fabra-García, J. Bolscher, W. J. R. Stone, I. Bally, S. Dergan-Dylon, I. Kucharska, R. M. de Jong, M. de Bruijni, T. Bousema, C R. King, R. S. M. Gill, R. W. Sauerwein, J.-P. Julien, P. Poignard, M. M. Jore, Target-agnostic identification of human antibodies to *Plasmodium falciparum* sexual forms reveals cross stage recognition of glutamate-rich repeats. bioRxiv [Preprint] (2024); https://doi.org/10.1101/2023.11.03.565335.

20. K. T. Ko, F. Lennartz, D. Mekhaiel, B. Guloglu, A. Marini, D. J. Deuker, C. A. Long, M. M. Jore, K. Miura, S. Biswas, M. K. Higgins, Structure of the malaria vaccine candidate Pfs48/45 and its recognition by transmission blocking antibodies. *Nat. Commun.* **13**, 5603 (2022).

21. A. Fabra-García, S. Hailemariam, R. M. de Jong, K. Janssen, K. Teelen, M. van de Vegte-Bolmer, G.-J. van Gemert, D. Ivanochko, A. Semesi, B. M. Leod, M. W. Vos, M. H. C. de Bruijni, J. M. Bolscher, M. Szabat, S. Vogt, L. Kraft, S. Duncan, M. R. Kamya, M. E. Feeney,

P. Jagannathan, B. Greenhouse, K. J. Dechering, R. W. Sauerwein, C. R. King, R. S. M. Gill, T. Bousema, J.-P. Julien, M. M. Jore, Highly potent, naturally acquired human monoclonal antibodies against Pfs48/45 block *Plasmodium falciparum* transmission to mosquitoes. *Immunity* **56**, 406–419.e7 (2023).

22. C. E. Farrance, A. Rhee, R. M. Jones, K. Musiychuk, M. Shamloul, S. Sharma, V. Mett, J. A. Chichester, S. J. Streatfield, W. Roeffen, M. van de Vegte-Bolmer, R. W. Sauerwein, T. Tsuboi, O. V. Muratova, Y. Wu, V. Yusibov, A plant-produced Pfs230 vaccine candidate blocks transmission of *Plasmodium falciparum*. *Clin. Vaccine Immunol.* **18**, 1351–1357 (2011).

23. A. Katureebe, K. Zinszer, E. Arinaitwe, J. Rek, E. Kakande, K. Charland, R. Kigozi, M. Kilama, J. Nankabirwa, A. Yeka, H. Mawejje, A. Mpimbaza, H. Katamba, M. J. Donnelly, P. J. Rosenthal, C. Drakeley, S. W. Lindsay, S. G. Staedke, D. L. Smith, B. Greenhouse, M. R. Kamya, G. Dorsey, Measures of malaria burden after long-lasting insecticidal net distribution and indoor residual spraying at three sites in Uganda: A prospective observational study. *PLOS Med.* **13**, e1002167 (2016).

24. R. W. Sauerwein, J. Plieskatt, M. Theisen, 40 years of Pfs48/45 research as a transmission-blocking vaccine target of *Plasmodium falciparum* malaria. *Am. J. Trop. Med. Hyg.* **107**, 22–26 (2022).

25. E. T. Bekkering, R. Yoo, S. Hailemariam, F. Heide, D. Ivanochko, M. Jackman, N. I. Proellochs, R. Stoter, G.-J. van Gemert, A. Maeda, T. Yuguchi, O. T. Wanders, R. C. van Daalen, M. R. Inklaar, C. M. Andrade, P. W. T. C. Jansen, M. Vermeulen, T. Bousema, E. Takashima, J. L. Rubinstein, T. W. A. Kooij, M. M. Jore, J.-P. Julien, Cryo-EM structure of endogenous Pfs230:Pfs48/45 complex with six potent antibodies reveals mechanisms of malaria transmission-blocking activity. *Immunity*, S1074-7613(25)00425-X (2025).

26. M. H. Dietrich, J. Chmielewski, L. J. Chan, L. L. Tan, A. Adair, F. M. T. Lyons, M. Gabriela, S. Lopaticki, T. A. Dite, L. F. Dagley, L. Pazzagli, P. Gupta, M. Kamil, A. M. Vaughan, R. Rojrung, A. Abraham, R. Mazhari, R. J. Longley, K. Zeglinski, Q. Gouil, I. Mueller, S. A. Fabb, R. Shandre-Mugan, C. W. Pouton, A. Glukhova, S. Shakeel, W. H. Tham, Cryo-EM

structure of endogenous *Plasmodium falciparum* Pfs230 and Pfs48/45 fertilization complex. *Science* **389**, eady0241 (2025).

27. W. K. Tang, C. H. Coelho, K. Miura, B. C. Nguemwo Tentokam, N. D. Salinas, D. L. Narum, S. A. Healy, I. Sagara, C. A. Long, P. E. Duffy, N. H. Tolia, A human antibody epitope map of Pfs230D1 derived from analysis of individuals vaccinated with a malaria transmission-blocking vaccine. *Immunity* **56**, 433–443.e5 (2023).

28. D. Ivanochko, A. Fabra-García, K. Teelen, M. van de Vegte-Bolmer, G. J. van Gemert, J. Newton, A. Semesi, M. de Bruijni, J. Bolscher, J. Ramjith, M. Szabat, S. Vogt, L. Kraft, S. Duncan, S. M. Lee, M. R. Kamya, M. E. Feeney, P. Jagannathan, B. Greenhouse, R. W. Sauerwein, C. Richter King, R. S. MacGill, T. Bousema, M. M. Jore, J. P. Julien, Potent transmission-blocking monoclonal antibodies from naturally exposed individuals target a conserved epitope on *Plasmodium falciparum* Pfs230. *Immunity* **56**, 420–432.e7 (2023).

29. M. H. Dietrich, M. Gabriela, K. Reaksudsan, M. W. A. Dixon, L. J. Chan, A. Adair, S. Trickey, M. T. O'Neill, L. L. Tan, S. Lopaticki, J. Healer, S. Keremane, A. F. Cowman, W. H. Tham, Nanobodies against Pfs230 block *Plasmodium falciparum* transmission. *Biochem. J.* **479**, 2529–2546 (2022).

30. C. H. Coelho, W. K. Tang, M. Burkhardt, J. D. Galson, O. Muratova, N. D. Salinas, T. L. Alves e Silva, K. Reiter, N. J. MacDonald, V. Nguyen, R. Herrera, R. Shimp, D. L. Narum, M. Byrne-Steele, W. Pan, X. Hou, B. Brown, M. Eisenhower, J. Han, B. J. Jenkins, J. Y. A. Doritchamou, M. G. Smelkinson, J. Vega-Rodríguez, J. Trück, J. J. Taylor, I. Sagara, S. A. Healy, J. P. Renn, N. H. Tolia, P. E. Duffy, A human monoclonal antibody blocks malaria transmission and defines a highly conserved neutralizing epitope on gametes. *Nat. Commun.* **12**, 1750 (2021).

31. W. J. R. Stone, J. J. Campo, A. L. Ouédraogo, L. Meerstein-Kessel, I. Morlais, D. da, A. Cohuet, S. Nsango, C. J. Sutherland, M. van de Vegte-Bolmer, R. Siebelink-Stoter, G. J. van Gemert, W. Graumans, K. Lanke, A. D. Shandling, J. V. Pablo, A. A. Teng, S. Jones, R. M. de Jong, A. Fabra-García, J. Bradley, W. Roeffen, E. Lasonder, G. Gremo, E. Schwarzer, C. J. Janse, S. K. Singh, M. Theisen, P. Felgner, M. Marti, C. Drakeley, R. Sauerwein, T. Bousema,

M. M. Jore, Unravelling the immune signature of *Plasmodium falciparum* transmission-reducing immunity. *Nat. Commun.* **9**, 558 (2018).

32. M. Theisen, W. Roeffen, S. K. Singh, G. Andersen, L. Amoah, M. van de Vegte-Bolmer, T. Arens, R. W. Tiendrebeogo, S. Jones, T. Bousema, B. Adu, M. H. Dziegiel, M. Christiansen, R. Sauerwein, A multi-stage malaria vaccine candidate targeting both transmission and asexual parasite life-cycle stages. *Vaccine* **32**, 2623–2630 (2014).

33. J. Rappsilber, M. Mann, Y. Ishihama, Protocol for micro-purification, enrichment, pre-fractionation and storage of peptides for proteomics using StageTips. *Nat. Protoc.* **2**, 1896–1906 (2007).

34. J. Cox, M. Mann, MaxQuant enables high peptide identification rates, individualized p.p.b.-range mass accuracies and proteome-wide protein quantification. *Nat. Biotechnol.* **26**, 1367–1372 (2008).

35. Y. Perez-Riverol, J. Bai, C. Bandla, D. García-Seisdedos, S. Hewapathirana, S. Kamatchinathan, D. J. Kundu, A. Prakash, A. Frericks-Zipper, M. Eisenacher, M. Walzer, S. Wang, A. Brazma, J. A. Vizcaíno, The PRIDE database resources in 2022: A hub for mass spectrometry-based proteomics evidences. *Nucleic Acids Res.* **50**, D543–D552 (2022).

36. B. C. van Schaijk, M. R. van Dijk, M. van de Vegte-Bolmer, G. J. van Gemert, M. W. van Dooren, S. Eksi, W. F. G. Roeffen, C. J. Janse, A. P. Waters, R. W. Sauerwein, Pfs47, paralog of the male fertility factor Pfs48/45, is a female specific surface protein in *Plasmodium falciparum*. *Mol. Biochem. Parasitol.* **149**, 216–222 (2006).

37. W. J. Stone, M. Eldering, G. J. van Gemert, K. H. W. Lanke, L. Grignard, M. G. van de Vegte-Bolmer, R. Siebelink-Stoter, W. Graumans, W. F. G. Roeffen, C. J. Drakeley, R. W. Sauerwein, T. Bousema, The relevance and applicability of oocyst prevalence as a read-out for mosquito feeding assays. *Sci. Rep.* **3**, 3418 (2013).

38. J. Ramjith, M. Alkema, J. Bradley, A. Dicko, C. Drakeley, W. Stone, T. Bousema, Quantifying reductions in *Plasmodium falciparum* infectivity to mosquitos: A sample size

calculator to inform clinical trials on transmission-reducing interventions. *Front. Immunol.* **13**, 899615 (2022).

39. C. Steentoft, S. Y. Vakhrushev, H. J. Joshi, Y. Kong, M. B. Vester-Christensen, K. T. B. G. Schjoldager, K. Lavrsen, S. Dabelsteen, N. B. Pedersen, L. Marcos-Silva, R. Gupta, E. Paul Bennett, U. Mandel, S. Brunak, H. H. Wandall, S. B. Levery, H. Clausen, Precision mapping of the human O-GalNAc glycoproteome through SimpleCell technology. *EMBO J.* **32**, 1478–1488 (2013).

40. R. Gupta, S. Brunak, Prediction of glycosylation across the human proteome and the correlation to protein function. *Pac. Symp. Biocomput.*, 310–322 (2002).
